# Reconstructing cancer drug response networks using multitask learning

**DOI:** 10.1186/s12918-017-0471-8

**Published:** 2017-10-10

**Authors:** Matthew Ruffalo, Petar Stojanov, Venkata Krishna Pillutla, Rohan Varma, Ziv Bar-Joseph

**Affiliations:** 1 Computational Biology Department, School of Computer Science, Carnegie Mellon University, Pittsburgh, PA USA; 2 Machine Learning Department, School of Computer Science, Carnegie Mellon University, Pittsburgh, PA USA; 3 Electrical and Computer Engineering, School of Computer Science, Carnegie Mellon University, Pittsburgh, PA USA

**Keywords:** LINCS, TCGA, Machine learning

## Abstract

**Background:**

Translating in vitro results to clinical tests is a major challenge in systems biology. Here we present a new Multi-Task learning framework which integrates thousands of cell line expression experiments to reconstruct drug specific response networks in cancer.

**Results:**

The reconstructed networks correctly identify several shared key proteins and pathways while simultaneously highlighting many cell type specific proteins. We used top proteins from each drug network to predict survival for patients prescribed the drug.

**Conclusions:**

Predictions based on proteins from the in-vitro derived networks significantly outperformed predictions based on known cancer genes indicating that Multi-Task learning can indeed identify accurate drug response networks.

**Electronic supplementary material:**

The online version of this article (doi:10.1186/s12918-017-0471-8) contains supplementary material, which is available to authorized users.

## Background

While several large scale efforts have recently focused on profiling the genome and transcriptome of cancer patients [1, 2], it is obviously much harder to test a large number of potential perturbations (gene knock downs, different drugs) for such individuals. Instead, recent efforts aimed at inferring cellular response networks that are activated by such perturbations have utilized in vitro cell lines. Such cell lines have now been derived for several different types of cancer [3–7] and these have been extensively used to study potential treatments and mutants. A recent example of such large scale cell line based project is the Library of Integrated Network-Based Cellular Signatures (LINCS) [8] a NIH-sponsored project that aims to characterize gene expression changes and other cellular processes under various perturbations, for the purpose of gaining better insight into biological networks.

While the tens of thousands of LINCS expression experiments provide valuable information regarding the response of specific cell lines to drugs, modeling the signaling and regulatory response networks using this data remains a challenge. Such models are critical if we intend to use the experimental results to improve the diagnosis and prognosis analysis of individuals. While cell lines and patient expression are likely to be different due to several technical issues [9], the underlying networks activated by the drugs are likely to be similar and so the ability to reconstruct these networks opens the door for using these drug specific experiments to tailor treatments to individuals.

Over the last decade several methods have been developed for reconstructing molecular response networks [10–13]. These methods often combine general interaction and sequence data with condition specific data to model pathways that are activated as part of the biological process being studied. While such methods have been successful in many cases, they face the same set of challenges that many other high throughput analysis methods face: the need to fit a large number of parameters using relatively few data samples. In the context of network reconstruction these parameters correspond to the presence of a specific protein (node) or an edge in the network [14], the direction of edges that are used [15, 16], the impact of an edge on a protein etc. Since the number of parameters is often greater than the effective number of input values this can lead to overfitting even when analyzing relatively large datasets for a specific condition [17, 18].

So far, most modeling methods are applied to reconstruct networks for a single condition/cell type at a time. One possible direction to overcome the data scarcity problem is to utilize datasets from *other*, similar, conditions when trying to reconstruct networks for a specific condition. Consider for example the task of reconstructing drug response networks in prostate cancer cell lines. Assume that in addition to the prostate cancer data we also have response data from breast cancer cell lines. Since breast cancer is likely utilizing some of the same pathways active in prostate cancer cell lines, at least some of the response is shared between the two cell types. Similarly, it is likely that we would observe at least some overlap in the activated regulatory modules between these cancer cell types. Indeed, such common expression activation has been widely observed in practice. For example, early work in yeast indicated that several genes are responding in a similar way to different types of stresses [19]. Similarly, we and others have shown that immune response to similar viruses (for example, different variants of flu) activates a large overlapping set of genes [13, 20], again supporting the idea of joint analysis of such data.

Given these similarities, a possible strategy to model response networks is to develop methods that can combine information across cell types while still generating cell type specific networks. Methods that attempt to perform such joint analysis are often referred to as *multi-task learning* algorithms [21] and have been applied to a number of different computational biology problems, most notably protein classification [22] and GWAS analysis [23, 24]. More recently, we have introduced MT-SDREM [13], the first multi-task method for learning dynamic regulatory networks for multiple immune responses. MT-SDREM combines a graph orientation method with Hidden Markov models (HMMs) to simultaneously reconstruct networks for several flu variants. However, while MT-SDREM was shown to successfully reconstruct these flu response networks, it suffers from a number of problems that limit its usability and effectiveness. First and foremost, MT-SDREM requires as input time series gene expression data. This obviously greatly limits its usability since most gene expression data is static [25]. In addition, MT-SDREM is actually optimizing two separate target functions (one for the HMM and the other for a graph orientation problem) making it very hard to converge to a joint (locally) optimal solution. Finally, MT-SDREM requires users to specify the set of sources (starting points for the reconstructed pathways). While such sources are known in some cases (for example, for flu) there are many cases in which they are either not known or not fully known which again limits its usability. Other work such as [26] focuses on multi-task learning of subnetworks in a protein interaction network, using somatic mutation data, expression data, and proteomic data, identifying common pathways between breast cancer patients. However, this method does not directly identify *regulatory* relationships, such as those linking transcription factors to differentially expressed genes, and it is difficult to identify *de novo* pathways due to the limitations of physical protein interaction networks.

To address these issues we developed a new multi-task learning framework to reconstruct signaling and regulatory networks that are activated in drug response experiments. We used our method to integrate a large number of gene expression experiments across multiple cancer types from LINCS to reconstruct drug response networks. By simultaneously analyzing several types of cancers for each drug we were able to improve upon networks constructed by analyzing each cancer type separately and upon the analysis of gene expression alone. Additional analysis of these networks identifies both, key proteins joint between cancer cell types as well as cancer type specific proteins. Finally, we used the top genes identified by our method for specific cancer drugs to predict patient response to that drug. As we show, by focusing on the networks activated in the vitro studies we were able to greatly improve patient survival predictions following treatment with a specific drug when compared to using known cancer genes.

## Results

**Fig. 1:**
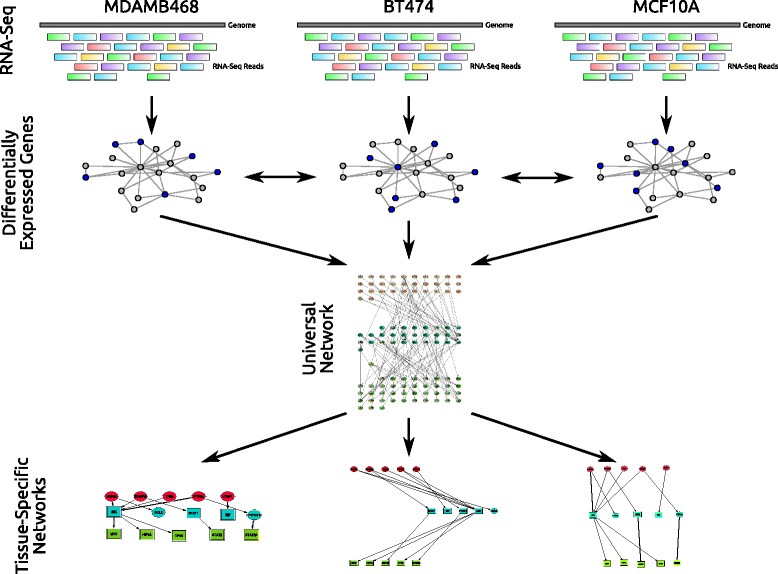
Overview of the multi-task learning method. RNA-Seq data from drug response experiments in different cell lines or cancer types (top) is used to select pathways linking source proteins to DE genes in general protein-protein and protein-DNA interaction networks (second row). Reconstructed networks are constrained by encouraging pathways that are shared across different cancer types leading to a general network (third row) that captures the common pathways activated during the response. In addition to the general network, cell type specific networks are also identified (bottom) and these can help identify tissue specific proteins and explain differences in response of certain cancer types when treated with the same drug

We developed a new Multi-Task (MT) learning formulation for integrating expression experiments across different types of drugs administered to cancer cell lines (Fig. 1). The goal of the method is to recover the pathways that are activated following treatment with a specific drug. To identify such pathways we define a target function that aims to explain the observed differentially expressed (DE) genes following treatment with the drug using paths that connect sources (potential drug targets) and DE genes in the network. Sources are either proteins that are known to directly interact with the drugs or proteins whose knock-out leads to expression profiles that are very similar to those observed for the specific drug treatment of the same cell (Methods). DE genes are selected separately for each drug / cell line combination. Following our assumption that most drugs activate the same pathways across different tissues / cancer types, the joint (MT) learning framework is used to constrain the set of paths in the resulting networks by encouraging compact solutions that are shared across the different tasks (cancer types). We developed a greedy algorithm for learning and inference in this model. Thus, while the learning is performed simultaneously for all types of cancer, we still obtain a specific network for each of the different cancer types. Next, we rank the top proteins in each of the cancer specific networks based on the number of selected paths that go through them (Methods) and analyze their relevance to the tissue and cancer with which they are associated by the MT analysis.

### Data and cell types

To test our method and to apply it to study drug response in cancer cell lines we used data from the LINCS consortium. One instance of the LINCS project is the L1000 (LDS-1191) dataset, which consists of Luminex gene expression data for 978 landmark genes (which have been selected based on the ability to infer expression values for all other genes from this set, see http://www.lincsproject.org/LINCS/data). These genes were profiled in multiple cell lines following treatment by several chemical reagents. Here we focus on experiments related to 12 known cancer drugs in 8 different types of cells. As mentioned above, we also used this data to determine sources for drugs and targets for TFs. Overall we have used more than 11,000 expression experiments for reconstructing the networks presented below.

As for cell types, we selected cell types based on overlap with drugs of interest and the availability of expression data from gene knockouts and administration of these drugs. In LINCS there are 52 breast cancer cell lines, 8 prostate cancer cell lines and 56 melanoma cell lines; we therefore tested our method using cell lines from breast cancer (MCF7), prostate cancer (PC3, VCAP) and melanoma (A375), as well as a non cancer cell line (HA1E) and data from primary tissue experiments. For drugs, we studied drugs that are used to treat multiple tumor types (methotrexate, clofarabine, idarubicin, paclitaxel, bicalutamide, bortezomib) as well as drugs that have been developed to specifically treat prostate cancer (disulfiram, docetaxel, ketoconazole, vinblastine, doxorubicin, metformin).

### Evaluation and comparison of the multi-task learning framework

We first tested our method by comparing its ability to correctly recover cancer related genes and pathways with results from the commonly used single task analysis for the same input data. For this we ran our method both in the multi-task setting and in a single task setting which uses the same objective function *without* the multi-task regularization terms (last term of Eq. 2). We have also compared the network based analysis results (both multi and single tasks) with the standard DE gene analysis methods that is commonly used, both for each experiment on its own and for a joint ranked list of DE genes [27]. For these comparisons, we ran our multi-task learning method on three separate sets of cells:

1. Normal (non-cancer) cells: A normal cell line (HA1E) and data from primary tissue (NPC). These were used as control experiments.
2. Different cancer cell lines: A breast cancer (MCF7) and a prostate cancer (PC3) cell line.
3. Two different prostate cancer cell lines: PC3 and VCAP which should be the most similar in their responses.

To reduce the effects of highly connected nodes in the network that tend to appear as top ranking genes for all drugs / cells we filtered the resulting set of top ranked genes for each run (both in the multi-task and the single tasks) to remove genes that appear in the top 100 for a random set of 20 non cancer drugs (Additional file 1). For the cancer cells we also performed the DE gene analysis using the *z*-scores derived by LINCS. We used a number of complimentary datasets for validation: the cancer gene census (CGC), GO, and MSIGDB genesets (Methods).

The results are summarized in Table 1 (see Additional file 1 results for complete tables with a breakdown for each of the drugs). For each set of cells we present the average overlap with validation genes/genesets across the six drugs. We also evaluate the gene rankings produced by our multi-task framework using the normalized discounted cumulative gain (nDCG) measure [28, 29], with results shown in Additional file 1: Figure S7 and described in Additional file 1: “NDCG Measure” section. We see that these results are comparable to those in Table 1.

**Table 1:** Comparison of different gene and network analysis methods for the reconstruction of drug response networks

Control	MT	Cell 1	Cell 2	MT-Diff	Diff-Cell1	Diff-Cell2
CGC	13.33	8.5	8.66	6.33	4.66	6.5
GO	72.33	33.66	41.5	47.66	43.16	29.5
Oncogenic	7.66	3	3.33	10.33	10.33	4.83
Breast & Prostate						
CGC	14.66	8.33	10	2.66	3.33	1.83
GO	77.5	70.66	64.83	18.16	25.66	18.33
Oncogenic	8.66	4.33	5.16	2.66	2.5	2.5
Prostate						
CGC	15	10.16	10.33	3.33	2.33	3.83
GO	82.33	85.83	88.66	23	26.83	18.5
Oncogenic	11	8.33	7.66	3	4.5	3.16

Values for each gene set and learning method denote the average number of genes (across six drugs) selected by each method which are also contained in the corresponding validation set. MT: multi-task, Cell 1, Cell 2: single task analysis (cell type based) for the two cells. “Diff” columns show genes selected only by differential expression (DE); MT-Diff: DE set for the two cell types, selecting genes that are differentially expressed in both cells, Diff-Cell1/2: cell type specific DE set

As can be seen in Table 1, comparing the results for the three analyses, we see that overall using the network structure improves upon methods that are only using gene expression data. Within the network reconstruction comparisons, the multi-task formulation performs better than selecting genes by differential expression alone in 8 of the 9 validation sets, and the single validation set in which differentially expressed genes outperform multi-task genes is oncogenic gene sets in non-cancer cells. Genes selected by the multi-task formulation likewise outperform those selected by the single-task formulation in 8 of the 9 validation sets/cell types studied. This increased performance holds true even for the normal cell lines and a possible reason may be the fact that these are all cancer drugs and so the pathways triggered by them are likely similar between the two non cancer cell lines as well. However, the performance is clearly better overall for cancer cell lines when compared to non cancer cell lines (in terms of the number of relevant genes and sets identified) and within the two cancer cell line sets, the performance for the prostate set is the best for two of three validation sets (CGC and oncogenic gene sets). This result agrees well with our assumptions regarding the advantages of multi-task learning. The more similar the tasks (in this case the same cancer type vs. two different types of cancer) the more likely it is that the pathways activated by the different drugs should be the same. Thus, the results in Table 1 validate both the usefulness of multi-task learning and its ability to correctly identify relevant cancer genes in drugs response experiments.

### Shared pathways in cancer drug responses

We next applied the multi-task learning framework to characterize the response of cancer cell lines to general cancer drugs. For this, we used three different cell types: melanoma (A375) breast (MCF7) and prostate cancer (PC3). As before, in Table 2 we observe that for the three metrics described above (averaged across six drugs), multi-task learning performs better than single-task, in all but one case (prostate cancer, when comparing to the MSIGDB oncogenic genesets).

**Table 2:** Results for breast cancer, prostate cancer and melanoma

	MTL	Breast	Prostate	Melanoma
CGC	28.66	22.33	22.66	21.16
GO	222	179.66	209.66	189.66
Oncogenic	14.16	9.3	14.83	4

Values for each gene set and learning method denote the average number of genes (across six drugs) selected by each method which are also contained in the corresponding validation set

**Fig. 2:**
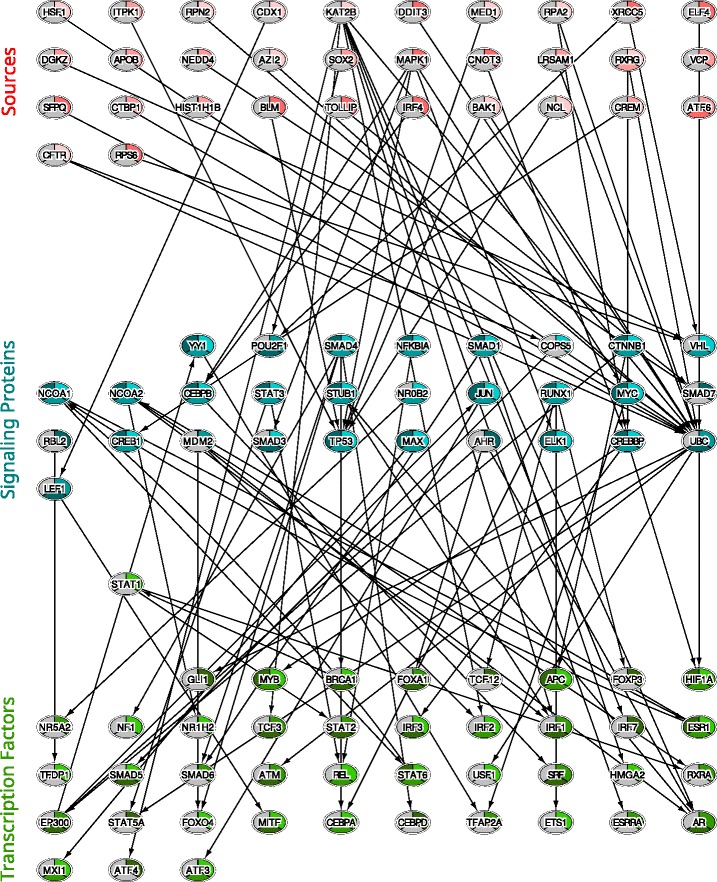
A merged network for the output of multi-task learning using data from breast cancer (lightest shade), prostate cancer (medium shade), and melanoma (darkest shade). Top nodes (red shades): Sources. These proteins are either known to interact with the drugs we tested or determined to be sources using the correlation analysis between drug expression response and KO response as described in Methods. Middle nodes (blue shades): Signaling proteins. These proteins are determined to belong to key pathways connecting sources and TFs. Bottom nodes (green shades): TFs. These proteins regulate a large subset of the DE genes in the different cell types following treatment with the drugs being tested. Note that while sources tend to be cell type specific, most signaling and TF proteins are shared between two or all three cell types indicating that several of the response pathways may be shared between the different cancer types

In order to visualize our results for this analysis, we have merged the pathways across drugs and cell types in order to represent them as one network (Additional file 1). Our network representation (Fig. 2) consists of sources (labeled in red), intermediate nodes (labeled in cyan) and transcription factors (labeled in green). Several important genes in the network are known cancer regulators or targets. These include *TP53*, a tumor suppressor protein involved in DNA repair and apoptosis which is known to be significantly mutated in all three cancer types [30]. As determined by our reconstructed network, *TP53* has been shown to interact with *BRCA1* [31], which is one of the TFs that were significantly prevalent in the pathways of two of the tumor types (breast and prostate cancer). *BRCA1* is involved in regulating cell cycle control and DNA repair and is known to pose a hereditary risk for breast cancer. *ATM*, another gene that we identified as significant, is a serine / threonine kinase known to activate important DNA-repair genes upon double-strand DNA breaks. This tumor-suppressor gene is known to be significantly mutated and deactivated in CLL [32]. A study has also shown that it is a risk allele for breast cancer [33].

Overall, we observe a convergence process in cancer drug response pathways for the cell lines being studied. While most sources identified by multi-task learning using these three types of cells tend to be cell type specific (i.e. different direct targets for the different types of cells), the down stream pathways that are activated are much more similar among these cell types. Specifically, unlike sources, most signaling and TF proteins are shared between two, or all three cell types. This may result from the target function maximized by the MT method which encourages common pathways between the different cell lines. However, the fact that such pathways are identified may also indicate that while different drugs target different proteins, their down stream effects are shared between the different cancer types.

### Cell type specific genes

The above discussion has focused on pathways and genes that are common to the different cell types. We next performed an analysis to rank genes by tissue specificity (Additional file 1). Such genes may be of interest since they may explain why some drugs work on a subset of cancer types but not on the rest. Since the inclusion of cell type specific genes in the network is penalized by the objective function (because they are only used for one cell type) those that are still selected need to be able to explain key aspects of the cell type specific response to warrant their inclusion.

Table 3 presents several of the top cell type specific genes for each of the cell lines we tested. Interestingly, many of the top-ranked genes have been implicated in their respective tissue types. *HDAC3* (ranked 2nd for breast cancer) is a histone deacetylase (HDAC), a family of enzymes that regulates gene expression by interacting with histones. These enzymes have been shown to be associated with estrogen receptor (ER) [34], and HDAC inhibitors have been shown to be effective in the treatment of breast cancer. *MED1* (4th, breast cancer) has been shown interact with ER in alpha-positive breast cancer tumors [35]. *GNAS* (5th) was identified as a breast cancer driver [36].

**Table 3:** Recurrent Genes for Breast Cancer, Prostate Cancer and Melanoma

Tumor type	Gene	Potential role
Breast cancer	*MED1* [35]	Co-activator with Her2 of estrogen
		receptor (ER) in *α*-positive tumors.
	*HDAC3* [34]	Histone deacetylase involved in the
		oncogenic tumorigenesis of breast
		cancer.
	*WEE1* [63]	Inhibition of *WEE1* causes increased
		cell death in breast cell lines
	*GNAS* [36]	*GNAS* locus identified as a driver in
		20q amplified breast cancer
	*CDKN1B* [64]	Associated with increased breast
		cancer risks
	*APP* [65]	Androgen-induced gene that
		promotes proliferation activity of
		breast cancer cells.
Prostate cancer	*PTPN6* [66]	Tumoral Prostate Shows Different
		Expression Pattern
	*PDGFRB* [37]	Blockade of PDGF signaling induced
		apoptosis in metastatic PCa cells.
	*TAB2* [67]	Deletion of MAP3K7 at 6q12-22 is
		associated with early PSA recurrence
	*SQSTM1* [68]	PCa cell lines have high p62/*SQSTM1*
		levels required for cell survival
	*COPS2* [69]	Alien interacts with the human
		androgen receptor and inhibits
		prostate cancer cell growth
	*CTBP1* [38]	Dysregulated expression of *CTBP1*
		plays an important role in prostate
		cancer progression
Melanoma	*TNFRSF10A* [70]	In vitro IFN- *β* and TRAIL/Apo2L
		combination treatment had more
		potent apoptotic effects
	*ZFP36* [40]	Loss of TTP represents a key event
		in the establishment of melanomas
	*PRKCD* [71]	Overexpression in BL6 murine
		melanoma cells inhibits the
		proliferative capacity in vivo
	*TLE1* [72]	Diagnostic Immunohistochemical
		Marker for Synovial Sarcoma
	*XRCC5* [39]	One of only two DNA repair and
		replication proteins which are
		prognostic for melanoma

For prostate cancer, *PDGFRB* (1st) is a growth factor whose signaling inhibition has been shown to induce apoptosis in metastatic prostate cancer cells [37]. The 5th ranked genes, *CTBP1*, was shown to inhibit proliferation in prostate cancer cell lines, suggesting a potential role as an oncogene [38].

In the case of melanoma, our top ranked gene was *XRCC5*, which is involved in double-strand break repair of DNA has been shown to be upregulated in metastatic melanoma patients with significantly worse prognosis [39]. Another high-ranked gene, *ZFP36* (2nd) inhibits proliferation of A375 melanoma cell lines when maintained at high levels [40].

**Fig. 3:**
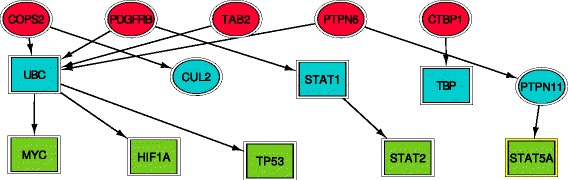
Tissue-specific pathways for prostate cancer. Tissue-specific prostate genes are shown as ellipses and other genes interacting with them are shown as squares. Red, sources, cyan, intermediate nodes, green, target nodes. *CUL2* (ranked 14th) and *PTPN11* (ranked 30th) were also on our list of prostate-specific genes

Figure 3 presents the prostate cancer specific pathways we obtained. In this figure we combine genes from Table 3 (labeled as ellipses) with other genes in the prostate-specific network (labeled as squares). Several of these pathways end in known cancer genes including *TP53* and *MYC*.

### Survival analysis using gene sets from the multi-task framework

So far we have focused on the analysis of in vitro data. However, a major question with respect to this data is how well such cell line based studies can inform us about in-vivo drug response. To address this question we combined the LINCS data and the results we obtained with data from The Cancer Genome Atlas (TCGA) [41]. TCGA contains gene expression and clinical data for 11,159 patients with several different types of cancer. Several of these patients were treated with drugs that were also profiled by LINCS and so we tested whether information extracted by our MT method from the LINCS data can be used to improve predictions regarding the way individuals would respond to specific drugs. Specifically, we have focused on three commonly prescribed drugs: paclitaxel, docetaxel, and doxorubicin which, combined were used by 1455 (13%) patients from TCGA (Additional file 1: Table S12). Note that other drugs studied in this multi-task framework were prescribed to too few patients to analyze in this way: methotrexate was the next most frequently prescribed medication, given to only 50 patients, and metformin was given to only 1 patient, as opposed to hundreds of patients given paclitaxel, docetaxel, and doxorubicin. For this analysis we downloaded mRNA expression data for these patients and used the expression values of the genes to learn a Cox regression model for predicting the 5 year survival of patients treated with each of these drugs. We compared five, equal sized, sets of genes for each of the drugs: (1) Top ranked genes from the multi-task learning method for that drug. Since we evaluate patients with several different types of cancer, for this analysis we combined the top ranked genes across all tissues into a single unordered gene set, and fit a model relating patient survival to expression of all genes in that set (Additional file 1). (2) Randomly sampled subsets of known general cancer genes from the COSMIC cancer gene census [42], and (3) Random sets of genes selected from all genes present in the expression data. (4) Genes selected by a single-task learning method applied to the same inputs as this multi-task method. (5) Genes selected by an elastic net Cox regression model, from all available genes in the gene expression data, with hyperparameter *λ* chosen to select at least as many genes as are present in the multi-task learning set for that drug. Additionally, we perform a separate Cox regression fit using genes in set 1 (identified by our multi-task method) but also including cancer/tissue type as a covariate, to evaluate the added effect of tissue type on prediction of patient survival. Since the multi-task gene set size is dependent on the specific drug (Additional file 1: Table S13), we evaluate the COSMIC, “all”, and single-task gene sets by randomly sampling subsets of genes equal in size to the multi-task gene set for each drug, and repeat this random sampling 100 times. Thus, for these sets we can also obtain confidence intervals.

**Fig. 4:**
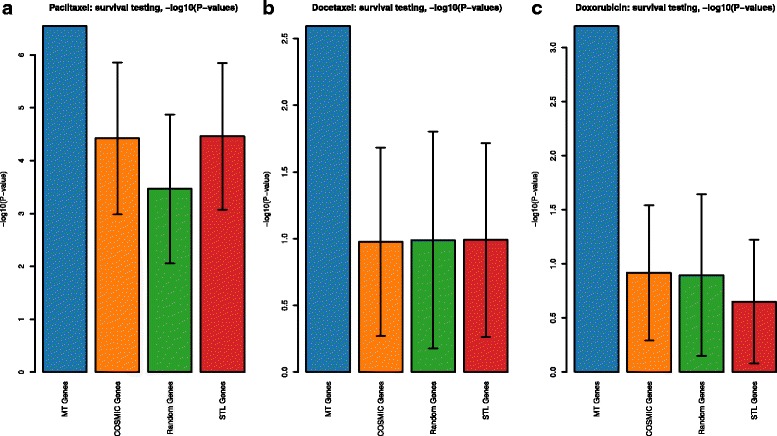
*P*-values for survival models fit using mRNA expression of genes in four sets: genes identified by the multi-task learning method for each drug, COSMIC cancer genes, all genes present in mRNA expression data, and single-task genes. For COSMIC, all genes, and single-task genes, 100 random subsets of available genes are chosen; each random subset contains the same number of genes as the multi-task set for a specific drug. Models are fit to a random training set chosen from 80% of patients, risk scores are calculated for training set and validation set samples, and the median risk in the training set is used as a threshold to divide validation set samples into two groups. *P*-values are computed from the difference in survival between the two groups of validation set samples. **a** shows results for paclitaxel, **b** shows docetaxel, **c** shows doxorubicin

**Fig. 5:**
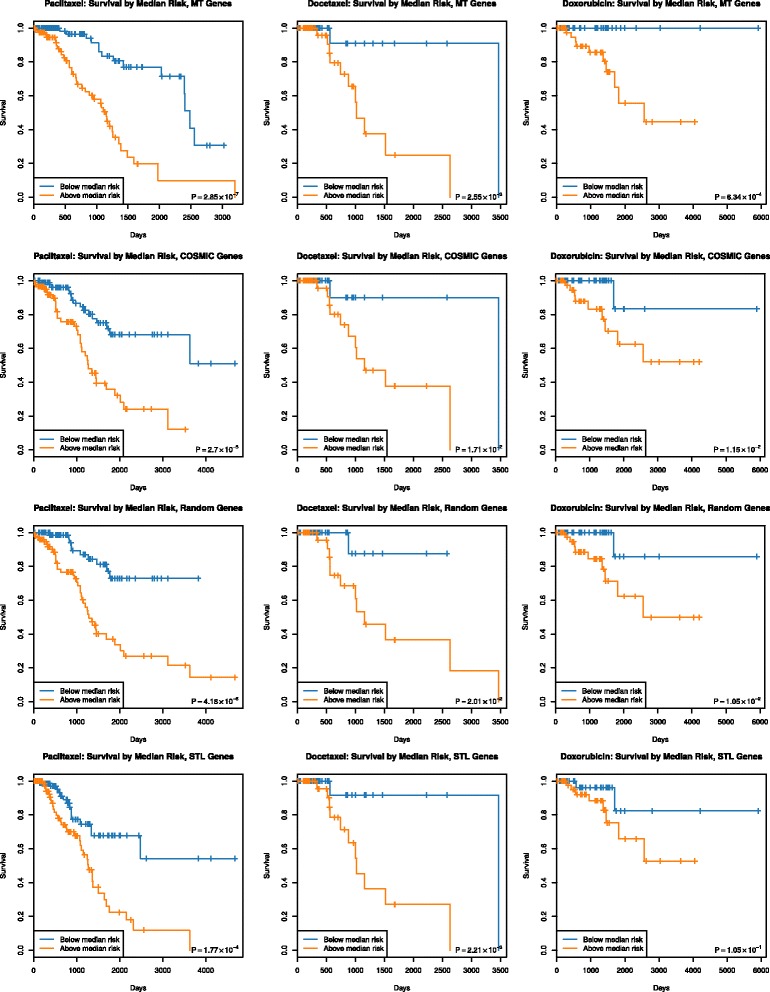
Kaplan-Meier survival curves for the survival analysis described in “Survival analysis using gene sets from the multi-task framework” section

We fit Cox regression models for each drug and gene set (multi-task, COSMIC subsets, elastic net selections, “all” subsets), relating the expression of these genes to the survival data for patients who were prescribed that drug. We perform an overall 80*%*/20*%* train/test split, and fit Cox models to the training set samples. We use the Cox model for each gene set to predict risk for both training and validation set samples, and use the median risk for training set samples as a threshold to divide the validation set samples into two groups, and compute *P*-values for the difference in survival between the two patient groups. For gene sets in which we randomly sample a subset of available genes (COSMIC, “all”, and single-task genes), we repeat this procedure 100 times, producing the *P*-value confidence intervals shown in Fig. 4. As expected, cancer specific genes from COSMIC are better at predicting survival when compared to random genes. However, drug specific genes identified by our method are significantly better than random selections from gene sets (COSMIC and “all” genes), and even outperform a survival-based gene selection using all 24,237 genes. In Additional file 1: Figure S1, we also see that inclusion of tissue type as a covariate does not consistently improve survival performance. Note that the patients included in our analysis were all those prescribed the drug and so represent several different types of cancer. Additionally, Fig. 5 shows Kaplan-Meier survival curves for these divisions of patients by each Cox model; plots for the multi-task genes for each drug are produced from the single Cox model described above. Plots for COSMIC, random, and single-task genes use Cox models from all 100 random samples of the appropriate gene sets; the overall threshold for the training set samples is chosen as a median-of medians: the median training set risk is computed for each of the 100 random samples of genes, and the overall threshold is the median of those values across training sets. The computed risk for each validation set sample is likewise computed as the median risk for that patient across the 100 Cox models, and the overall training set threshold is used to split the validation set samples. We see that despite the repeated sampling of other gene sets, genes selected by our multi-task method produce a better stratification of survival in validation set samples. For each drug, we also evaluate the robustness of these Cox regression models fit to expression of genes in that drug’s multitask gene set, across 5 cross-validation folds that stratify the set of patients who were given that drug. For each of these patient sets, we use expression of genes in that drug’s multitask gene set to fit Cox regression models, and compute a gene’s importance as the absolute value of its Cox regression coefficient in that cross-validation fold. We examine the consistency of these gene-wise importance measures between cross-validation folds by computing pairwise Spearman correlations between all $\binom {5}{2} = 10$ pairs of (absolute value) coefficient vectors. These correlation values are shown in Additional file 1: Figure S6. We see that these correlation measures range from 0.71 – 0.77 for docetaxel, with 65 genes identified by the MT method; 0.58 – 0.73 for doxorubicin, with 70 MT genes; and 0.66 – 0.83 for paclitaxel, with 113 MT genes. Thus, the in vitro LINCS data contains drug specific information that can be used across cancer types to predict drug efficacy much better than general onco-genes.

## Discussion

Several methods have been developed for reconstructing disease and drug response networks from gene expression data. However, even when integrated with general interaction datasets, these reconstruction methods tend to suffer from the scarcity data and the large parameter space which often leads to overfitting and other inaccuracies [43].

We developed a new multi-task learning framework for reconstructing signaling and regulatory response networks. Such methods provide the best of two worlds. On the one hand they can utilize related datasets when reconstructing the networks, reducing the data scarcity problem while on the other they still reconstruct specific response network for each condition/cell type. We designed an appropriate regularized objective function for this task and developed methods for efficiently searching for pathways that are commonly used across the tasks being modeled. Using LINCS datasets we were able to identify both sources and targets which were used as start and end points in the pathways we reconstruct.

Application of the multi-task learning framework to the analysis of drug responses in cancer cell lines allowed us to identify both common and cell type specific pathways. As expected, the common pathways contain many of the well known cancer genes as well as other genes involved in cell cycle and immune response activity [44]. Interestingly, the cell type specific pathway we obtained correctly assigned many proteins to their specific tissue. This represents an additional benefit of the multi-task learning framework. Since the usage of cell type specific pathways is penalized by the objective the only paths that would be included are those that are able to explain a large number of cell type specific targets. Thus, paths that are still included even though they are only assigned to one task represent key events in the cell type specific response. In contrast, it is very hard to identify such cell type specific pathways when performing the standard, single task, analysis since they can often score lower than those paths that contain general cancer response genes.

We used MSigDB gene sets to train the hyperparameters for our model. Thus, some of the improvement in terms of GO and census genes for MT vs. single task learning can be attributed to the overlap between the training and test data used. However, we only used breast cancer for training and so results for prostate and melanoma and their comparison to controls is still valid. In addition, as the survival analysis indicates the set of genes selected does not only improve the match with prior knowledge about cancer genes but also improves our ability to assess future outcomes which is an independent criteria.

While perturbation experiments such as those performed by LINCS can be carried out on cell lines, it is much harder to obtain molecular drug response data from patients. Most studies, including the large TCGA study, only provide a snapshot expression signature, usually obtained from the initial biopsy. Thus, a major challenge in translating genomic analysis to clinical application is to be able to predict, based on this initial sample, the response of the individual to the various treatment options available. Here we showed that by combining the in vitro cell line data with the patient specific RNA-Seq data we can greatly improve our ability to predict drug specific responses in several different types of cancer. The ability of the MT learning method to identify key proteins in the pathways that are the most responsive to the drug allowed it to correctly zoom in on these when training a regression model for each drug leading to much better results when compared to using general cancer genes. In addition, and unlike prior methods that relied on the patient expression data alone, the use of an external dataset (LINCS in this case) to train such model is likely to reduce overfitting since genes selected are not impacted by the specific way in which the clinical data was obtained [45].

While in this paper we looked for positive correlations between drugs treatment experiments and protein KD experiment to identify potential drug targets, the method can work with absolute correlations as well. In our case all drugs we looked at are known inhibitors and so we expected to see the same response direction for the drugs and their target KD experiments. However, if one is studying other types of drugs, including activators, using the absolute correlation may be a better choice.

## Conclusions

By using MT learning we were able to obtain accurate drug specific sets of genes from a large collection of in vitro expression experiments. The sets of genes identified by our method can be used to both, determine the tissue specificity of a response and the pathways it activates and to accurately predict survival when combining top ranked genes across tissues. The ability to integrate in vitro and in vivo data for such tasks is an important issue for efficiently translating experimental results to clinical tests.

## Methods

### General overview

A typical learning algorithm for classification or regression focuses on minimizing a loss function that is task specific. For example, learning a classifier for dogs is a different task than learning a classifier for cats and so a dog classifier would use a different set of parameters than a cat classifier. However, in many cases there exists domain-specific information that multiple tasks may share and that could potentially improve the set of parameters learned for *all* tasks. Evangelou et al. [46] and Caruana et al. [21] have shown that such approach works well for several cases of kernel regression and classification, as well as for other ML techniques. A common objective function for multi-task learning is as follows [46]:

1 $$\begin{array}{*{20}l} \text{arg\,min}_{w_{1}, \ldots, w_{2}} \left\{ \sum\limits_{i=1}^{C} L\left(y_{i}, f\left(\mathbf{w}_{i}^{T}x_{i}\right)\right) + \lambda_{1}\|w_{i}\|_{p} \right\} \\+ \lambda_{2} \sum\limits_{i=1}^{C} \sum\limits_{j=i+1}^{C}\|w_{i} - w_{j}\| \end{array} $$

Here, the first set of components of the objective function is the typical single task objective which seeks to identify the optimal (regularized) set of parameters to minimize a specific loss function. The second component is the multi-task addition. It is used to penalize differences between parameters assigned to each of the tasks, encouraging similar estimates across different tasks. Other formulations of the multi-task objective are also commonly used, including trace norm regularization [47–49]), joint feature learning [50], and robust multi-task feature learning [51], though these formulations all share the same goal of penalizing differences in model parameters between tasks. Multi-task learning is especially useful in cases where the training data for each specific task is limited, as is often the case in computational biology.

### A multi-task objective function for reconstructing drug response networks

We would like to formulate an objective function for reconstructing drug response networks in different cell types (where each cell type represents a task). These networks should (compactly) explain the observed expression response while encouraging sharing of nodes (proteins) and pathways across different tasks/cells types. Since many cancer drugs can successfully treat several types of cancers [52], we expect that in many cases different cancer cell types react to drugs using similar pathways. Thus, the multi-task learning approach allows us to utilize more data when constructing drug response networks while at the same time it can still identify cell type specific pathways.

We assume that for each drug and cell type we have a list of potential paths and that our goal is to select among these paths the subset that are activated in the response. Each path links a source (a protein that may interact directly with the drug) and a target (DE genes following drug treatment). Below we discuss how sources, targets and potential paths are determined. Algorithms developed for reconstructing cell type specific response models attempt to identify pathways that lead from sources to targets in the network using the least amount of intermediates [10, 53]. Such pathways provide the most compact explanation for the observed response following treatment while at the same time highlight the intermediate nodes (TFs and signaling proteins) that contribute to the response observed. Our multi-task learning objective function aims to balance this requirement (compact explanation of the observed response for each cell type) with the goal of using similar pathways for *all* the different responses we are studying. An overview of the method is shown in Fig. 1.

We use the following notations to formally present the objective:

### Notation

- *C*: set of all conditions - in our case the cell lines for a particular drug experiment
- *T*_*c*_: set of targets of a condition *c*∈*C*
- $P_{c}^{t}$: set of paths connecting *c*∈*C* to target *t*∈*T*_*c*_, from protein interaction data
- *h*(*p*): weight of a path computed as the product of probabilities of edges in the path
- *S*_*c*_ subgraph of the network corresponding to all *paths* selected for a condition *c*.
- *S*: subgraph of the network containing the union of all paths **from all conditions** *c*∈*C*.
- *I*_*S*_(*p*): 1 if *p*∈*S* and 0 otherwise
- *n*(*p*_1_,*p*_2_): number of nodes common to paths *p*_1_,*p*_2_
- *N*(*S*): total number of nodes present in all paths contained in *S*
- $\mathcal {T}_{c}$: set of TFs of condition *c*∈*C*
- $\mathcal {P}_{c}^{tf}$: set of paths connecting *c*∈*C* to $tf\in \mathcal {T}$
- *TF*(*S*): set of transcription factors in the network induced by *S*
- *T*(*tf*): set of **all** predicted targets of a transcription factor *tf*
- *DE*(*c,tf*): set of **differentially expressed** targets of *tf* in condition *c*.

### Objective function

We optimize the following objective function:

2 $$ \begin{aligned}  \max_{S = \bigcup_{c \in C} S_{c}} \left\{\lambda_{1}\sum\limits_{c\in C}\sum\limits_{t\in T_{c}}I\left(\left|S_{c} \cap P_{c}^{t}\right|>0\right)\right\} \\ + \left\{\lambda_{2} \sum\limits_{c \in C} \sum\limits_{tf\in TF(S_{c})}\frac{|DE(c,tf)|}{|T(tf)|}\right\} \\ + \left\{\lambda_{3}\sum\limits_{c \in C}\sum\limits_{p\in S_{c}}h(p)\right\} - \left\{\lambda_{4}N(S)\right\} \\ + \left\{ \lambda_{5}\sum\limits_{\{p_{i},p_{j}\} \in S, i \neq j}n(p_{i},p_{j})^{\alpha}\right\} \end{aligned}  $$

We explain each term separately below:

1. Given a set of discovered paths *S*_*c*_ (current subnetwork of a given condition), the first term is the loss function for the individual network reconstruction task. This term encourages explanation of as many targets as possible by summing up the number of targets that are explained by the selected pathways.
2. The second and third terms are the regularization terms for the single tasks. The 2nd term penalizes the use of TFs for which a large fraction of their targets are not DE in that condition while the third penalizes for paths that do not have a high weight (see below for how we compute a weight for a path)
3. Finally, the last two terms in the objective are the multi-task regularization parts. The 4th term penalizes the size of the selected union of subnetworks for each condition $S = \bigcup _{c \in C} S_{c}$ in terms of the total number of nodes included in all pathways selected encouraging nodes that are shared between tasks. The last term similarly encourages the selection of shared paths between the tasks.

We optimize this objective function across cell lines, producing a unified model for each distinct drug.

### Learning and inference

The NP-hard set cover problem can be reduced to the objective function listed above by appropriately selecting *λ*_1_ and *λ*_3_ (the first term encourages the use of all elements while the third term penalizes the use of too many sets/paths). We thus developed a greedy algorithm to optimize our objective. The main point of the algorithm is the focus on TFs rather than on the target themselves. Since we assume that each target (DE gene) needs to be activated/repressed by a TF upstream, the selection of a set of paths can be reduced to the appropriate selected of a subset of TFs that, together, cover as many of the targets as possible while not connected to many non DE genes. For this, we greedily add and remove TFs to the set of selected paths for each task (*S*_*c*_) until the target function no longer increases. Note that there are often several paths that can link TFs to sources and we need to select at least one of them (which means also selection of all intermediate nodes) in order to include the TF in our solution set. The identity of the best path for each TF is a function of the other protein/TFs that are already included and so should be re-determined in each iteration of the greedy search. See Additional file 1 for the set of algorithms we use to rank paths for each iteration and for selecting the TFs to include in the resulting networks.

Beyond inference (i.e. the selection of paths) the objective function has five parameters (*λ*_*i*_, *i*∈{1,2,3,4,5}) which should be set. To determine values for these parameters, we used a training set of 9 drugs and determined accuracy based on significant overlap with the MSIGDB genesets. As can be seen in Additional file 1: Table S1, we observed good agreement between the values determined for these parameters for the different drugs we tested and used these values for the analysis described in Results.

### Network construction

We used general protein-protein and protein-DNA interaction data to obtain a superset of all possible pathways. Protein interaction data was obtained from [54–56]. Note that that data contains probabilities for each of the edges in the network based on the confidence in the type of experiment that identified the interactions and these were used to determine path weights using the method defined in [57]. Protein-DNA interactions are composed of a potential set of targets based on motif analysis [58]. In addition, we extend the list of potential TF targets using LINCS KD data in the following way. For each TF knock-down performed by LINCS we add the top *d* DE genes to the potential set of targets for that TF. We use *d*=100 in this study though other values produced similar results.

### Using LINCS data to identify sources

While the drugs we used in this study have known direct targets, these are probably not the only targets of the drugs. Indeed, it has been observed in many cases that drugs can directly activate other proteins that are not designated as their official targets (often referred to as side effects, [59]). Thus, ignoring these (unknown) drug targets will likely negatively influence the ability of our method to explain the observed expression response.

We have thus further expanded the list of potential targets for each drug (sources in our networks) by using a large number of knockdown (KD) expression experiments from LINCS, as recent work has shown that LINCS data can be reliably used for drug target identification [60]. We hypothesized that if a protein is a direct target of a specific drug, its expression KD profile will be similar to the expression profile observed after applying the drug. To identify such direct targets we compute the correlation between the expression response of every KD experiment and the drug response for each cell line/drug. We next rank proteins based on this correlation and select a subset of the *k* highest ranked ones as potential sources. For this paper we have used *k*=100 though other values of *k* we tested led to similar results (Additional file 1: Table S9, gene lists posted on supplementary website).

### Ranking genes and evaluating the resulting networks

For each cell type and each drug, we obtain a set of pathways *S*_*c*_ that start at a source protein (representing a direct drug target) and ends at a gene target, i.e. a gene that is DE following treatment with the drug. We use network flow analysis to prioritize the set of key nodes in the networks (Additional file 1).

Molecular networks are generally very difficult to validate since there often is no known ground truth. We thus rely on complementary datasets for validation. These include GO (the Gene Ontology) [61] and 189 oncogenic genesets from MSIGDB [62]. We also use a set of 572 known cancer genes from the Cancer Gene Census [42]. We examine the overlap between genes contained in our molecular networks for each drug, and genes in these validation sets for multiple cell lines/types: control, breast and prostate cells combined, and only breast cancer. This produces cross-drug measures of our method’s ability to identify genes that are known to be involved in biological processes of interest.

### Finding common and cell type specific genes

Using multi-task learning we can identify both, genes that are shared between all cells we are modeling as well as cell type specific genes. This latter set is of particular interest since these are genes that the algorithm decided to include in the cell type specific network even though such inclusion incurs a penalty since they cannot be used for the other types of cells. Thus, these genes are likely key players in the cell type specific response. To find genes that are designated as cell type specific across several conditions (drugs) we perform the following procedure:

1. We create a 3D matrix *M*_3_ of dimension *N*_*g*_×*N*_*d*_×*N*_*t*_, where the *N*_*g*_ is the number of genes in the union of top discovered genes for the drugs and cell types we are studying, *N*_*d*_ is the number of drugs, and *N*_*t*_ is the number of tissue types.
2. For each entry of this matrix we compute the inverse of the rank 1/*r* that this gene has in that drug and cell type’s ranked list in the single-task scenario (ranked list obtained as described above).
3. For each cell type, we add these scores across the dimension of the drugs, which yields a *N*_*t*_×*N*_*g*_ matrix *M*_2_ with new summed scores. For clarity, let *r*_*i*_ be the rank of a particular gene *g* for a particular cell type *c* and for the *i* ^th^ drug. The entry *s*_*g,c*_ of matrix *M*_2_ for gene *g* and cell type *c* is given by: $s_{g,c} = \sum _{i=1}^{N_{d}} \frac {1}{r_{i}}$. For each gene and each cell type, we take the summed score and divide it by the average of the summed scores for this gene for all cell types, to obtain a final score *f*_*g,c*_. This is given by: $f_{g,c} = \frac {s_{g,c}}{\frac {1}{N_{c}} \sum _{i=1}^{N_{c}} s_{g, i}}$.

This MT framework therefore balances these two seemingly competing objectives, on one hand it tries to find genes that are associated with all cancer types being modeled as these will score high for both networks, but on the other hand it still produces condition or cell type specific networks which include genes that are unique to this cell type. Genes that are cell type specific need to be more critical to the network than the shared genes – in other words, these genes must be essential for explaining the flow of information for a specific cell type. Thus, our method balances these two competing requirements by placing a higher bar on the inclusion of task specific genes while still allowing them to be selected if necessary.

## Additional file

Additional file 1 Supporting Information. **S1 Text**: Description of the inference algorithm for the multi-task objective function, learning parameters for the multi-task objective, method for ranking genes in the resulting networks, construction of the network figure, filtering of high-degree genes, and supplementary tables. (PDF 475 kb)

## Abbreviations

CGC: Cancer gene census
DE: Differentially expressed
GO: Gene ontology
GWAS: Genome-wide association studies
HMM: Hidden Markov model
KD: Knock-down (of a specific gene or protein)
LINCS: Library of integrated network-based cellular signatures
MSigDB: Molecular signatures database
MT: Multi-task
nDCG: Normalized discounted cumulative gain
TF: Transcription factor
TCGA: The cancer genome atlas
