# Supplementary material for: Reconstructing cancer drug response networks using multitask learning
Source: BMC Syst Biol. 2017 Oct 10;11:96. doi: 10.1186/s12918-017-0471-8 (PMC5635550; doi:10.1186/s12918-017-0471-8)
Supplement: Additional file 1 — Supporting Information. S1 Text: Description of the inference algorithm for the multi-task objective function, learning parameters for the multi-task objective, method for ranking genes in the resulting networks, construction of the network figure, filtering of high-degree genes, and supplementary tables. (PDF 475 kb) [file 12918_2017_471_MOESM1_ESM.pdf]

Reconstructing cancer drug response networks using multitask learning
(Supplementary Material)

Matthew Ruffalo[1,†], Petar Stojanov[1,†], Venkata Krishna Pillutla[3], Rohan Varma[3], and Ziv Bar-Joseph[1,2]

[1] *Computational Biology Department, School of Computer Science, Carnegie Mellon University, Pittsburgh, PA, USA*
[2] *Machine Learning Department, School of Computer Science, Carnegie Mellon University, Pittsburgh, PA, USA*
[3] *Electrical and Computer Engineering, Carnegie Mellon University, Pittsburgh, PA, USA*
[†] *These authors contributed equally to this work.*

Contents

1 LINCS Data Levels **1**

2 Inference Algorithms for the Multi-Task Objective Function **2**

3 Learning parameters for the Multi-Task Objective **2**

4 Ranking genes in the resulting networks **3**

5 Network Figure **3**

6 Filtering High-Degree Genes **3**

7 Survival Analysis **4**

8 NDCG Measure **11**

9 Supplementary Tables **11**

1 LINCS Data Levels

The LINCS project defines data levels analogous to those used by the TCGA project for microarray-based gene expression analysis. From `https://tcga-data.nci.nih.gov/docs/publications/tcga/datatype.html`, TCGA data levels are as follows:

1. Raw signals per probe, for each sample

2. Normalized signals per probe, for each sample

3. Expression calls per gene, for each sample

As described in the "L1000 mRNA profiling assay" section at `http://lincsportal.ccs.miami.edu/dcic-portal/`, level 4 data (differential gene expression) is defined via a z-scoring procedure, producing a vector of differential expression for each gene in each sample (where a sample here is a cell line under the conditions of perurbagen introduction, gene knockout, gene overexpression, or other experiment types). In this work we use these level 4 z-scores to rank genes.

2 Inference Algorithms for the Multi-Task Objective Function

To determine the most significant paths from sources to targets that maximize the objective, the algorithm considers the set of source-TF pairs (given by (c, tf)), and also considers a limited number of paths between these pairs. We focus on TFs because they represent the ending point of a path within a PPI network, and once we decide to include a TF in the set of paths, we also include all of its targets. The first step of algorithm 1 finds k best paths for each source–TF pair (out of all paths in the PPI network) using BFS.

Algorithm 1 Overall Algorithm

Search Space: For every (c, tf), find k best paths from the network with BFS
Search Procedure: Algorithm 2

Next, given the set of paths obtained by algorithm 2 we pick an ordering τ in a randomized fashion using importance sampling with a probability computed as follows; for each path for a given (c, tf) pair we compute a weight $\exp(-\frac{1}{|p|}(\sum_{e \in p} - \log(h(e))))$ where $h(e)$ is the probability of edge e in path p. The path weights correspond to the probability of the path controlled for its length. The weights are then averaged across all paths of p of a given pair (c, tf) to get a score for it, and the pair is sampled with this score. Thus we have an ordered list of (c, tf) pairs proportional to the average probabilities of their paths that we will sequentially explore, which forms our greedy algorithm described below.

We begin with an empty sub-network S. Next, we iterate over (c, tf) pairs based on the ordering above and add a path for each (c, tf) pair until the objective does not increase. This is repeated for each TF until S no longer changes.

Algorithm 2 Greedy Algorithm

Input: k paths for each (c, tf) pair, ordering τ of (c, tf) pairs
Output: set of paths, S

$S = \phi$
for (c, tf) in ordering τ **do**
  while S changes **do**
    Find best path p_1 from c to tf to add to S
    Find best path $p_2 \in S$ to remove from S
    `Add`(p_1, S), `Remove`(p_2, S), or leave S unchanged, whichever leads to the highest objective function
  end while
end for
return S

3 Learning parameters for the Multi-Task Objective

The objective function has five parameters which should be set. To set these values we used a training set of 9 or 6 drugs and determined accuracy based on significant overlap with the MSIGDB genesets. 4 of 9 drugs were used for the cross-validation procedure. Table S1 contains some sample output we obtained in deciding on parameter values:

Table S1. Sample Cross-validation output

Drug	α	λ_1	λ_2	λ_3	λ_4	Q_m
9	1	0.1	-0.5	0.1	5	38
9	1	0.1	-0.5	0.05	5	33
6	1	0.1	-1	0.1	5	9
6	1	0.1	-0.5	0.1	5	40
6	1	0.1	-0.5	0.05	5	35

4 Ranking genes in the resulting networks

For each cell type and each drug, we obtain a set of pathways S_c that start at a source protein (representing a direct drug target) and ends at a gene target, i.e. a gene that is DE following treatment with the drug. We use network flow analysis to prioritize the set of key nodes in the networks. For a gene g, we sum the number of the gene's occurrence in the set of pathways selected by the algorithm weighted by the probability of the path using the following function: $r = \sum_{p \in S} I(g \in p)h(p)$ where $h(p)$ is the probability of the path.

To rank proteins that are joint between tasks we intersect the top L proteins for each task (here we use $L = 200$ though similar results are obtained when using other numbers for the set of top proteins).

5 Network Figure

We construct the network in the following manner:

1. For all three conditions in the figure we first rank the sources, the intermediate nodes and the transcription factors according to a score equal to their respective number of occurrences in the network weighted by the probability of each path that they occurred in. We merge these rankings across drugs by summing these scores for each gene across drugs and then sorting. We took the top 30 genes for the source layer, top 70 for the intermediate layer and top 60 for the transcription factor layer.
2. For each ordered pair of genes, we also calculate a score for an edge between them by calculating the number of occurrences weighted by the probability of the path in which they occurred. These edge scores are also summed across drugs and conditions in order to have one set of edges.
3. We plot nodes that have edges with a score above a certain threshold (we set this threshold to be 45). We color each layer in different colors (red for sources, cyan for intermediate nodes and green for transcription factors).

6 Filtering High-Degree Genes

In order to control for high-degree genes making it to our top-ranked genes, when evaluating the performance of mulit-task learning, we used a list control genes that we used to filter our results. In order to create this list, we used a list of 30 randomly picked drugs (whose LINCS IDs are presented in Table S2 below) and ran single-task learning on them. This resulted in 30 ranked lists (obtained as described in the main text) which we combine by recomputing the ranks of all genes present in these lists into scores as follows: for each gene we take its inverse ranks in each of the 30 lists and sum them up. We then sort the genes according to these new scores.

Table S2. Drug IDs used in filtering high-degree genes

torin-2
K784-3188
BRD-K26257340
amperozide
thiothixene
IKK-2-inhibitor-V
SEW-00445
RO-15-4513
dephostatin
saclofen
BRD-K59946561
cinalukast
decafluorobutane
olvanil
PD-0325901
bisoprolol
JNJ-7706621
modafinil
BRD-K95253417
BRD-K39187410
BRD-K35961280
clofibric-acid
topotecan
FK-888
RAN-07
midodrine
BRD-K73700643
GBR-12935
salsolinol
androstanol

7 Survival Analysis

Figure S1 shows survival concordance plots; the first four columns in each plot show the same data as in the manuscript proper. The fifth column shows performance of suvival models using MT genes and tissue type together, the sixth shows elastic net Cox regression, and the seventh shows survival models trained only on tissue type (treating this as a categorical variable for the Cox regression fit).

Figure S2 shows overall Kaplan-Meier survival curves for patients with each cancer type. Figure S3 shows the distribution of cancer (tissue) type for the patients prescribed each drug used in survival analysis.

Figures S4 and S5 show Kaplan-Meier curves similar to Figure 5 in the main text. Figure S4 shows the same curves as Figure 5, with survival curves for each drug plotted in the same axes for easier comparison. Figure S5 shows Kaplan-Meier curves for additional analysis types (gene sets, regression methods, additional covariates) not included in Figure 5.

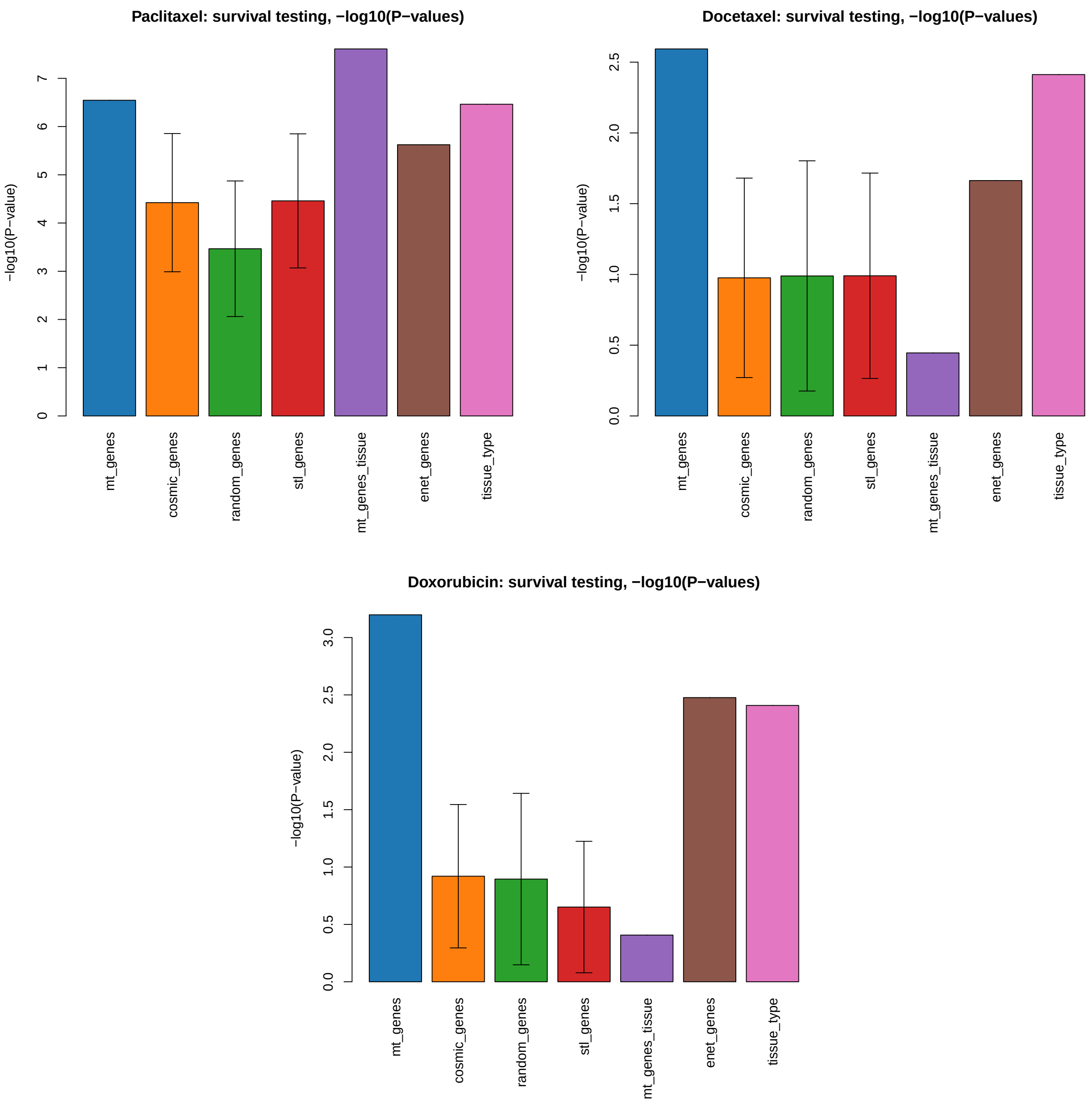

Figure S1. *P*-values for survival models fit using mRNA expression of genes in five sets: genes identified by the multi-task learning method for each drug, COSMIC cancer genes, all genes present in mRNA expression data, single-task genes, and genes selected by Elastic Net Cox regression. Additionally, separate analyses is performed with the addition of tissue type as a coviarate with expression of MT genes, and using tissue type alone to stratify patients. For COSMIC, all genes, and single-task genes, 100 random subsets of available genes are chosen; each random subset contains the same number of genes as the multi-task set for a specific drug. Models are fit to a random training set chosen from 80% of patients, risk scores are calculated for training set and validation set samples, and the median risk in the training set is used as a threshold to divide validation set samples into two groups. *P*-values are computed from the difference in survival between the two groups of validation set samples. (a) shows results for paclitaxel, (b) shows docetaxel, (c) shows doxorubicin.

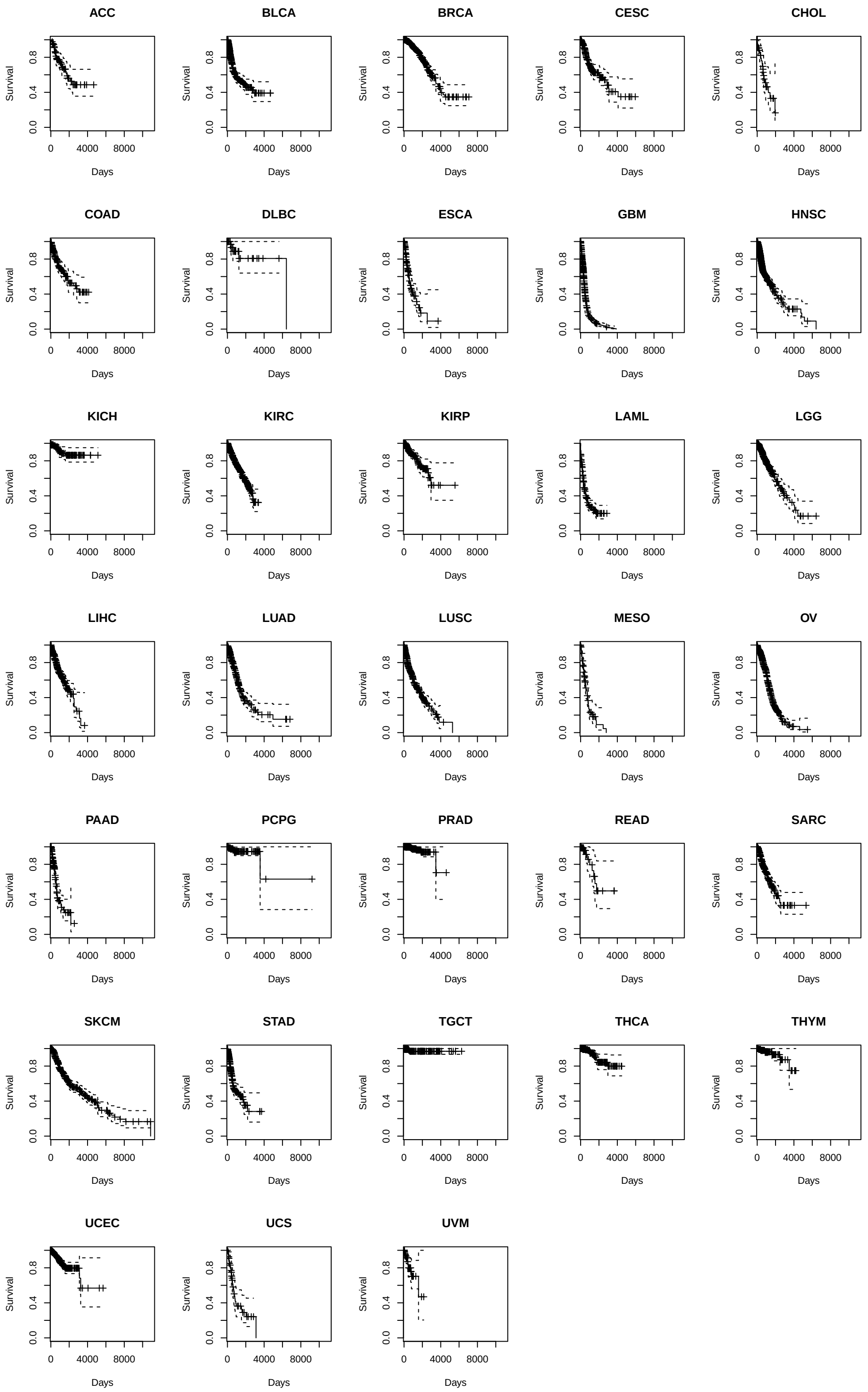

Figure S2. Overall Kaplan-Meier survival curves for patients diagnosed with each cancer type.

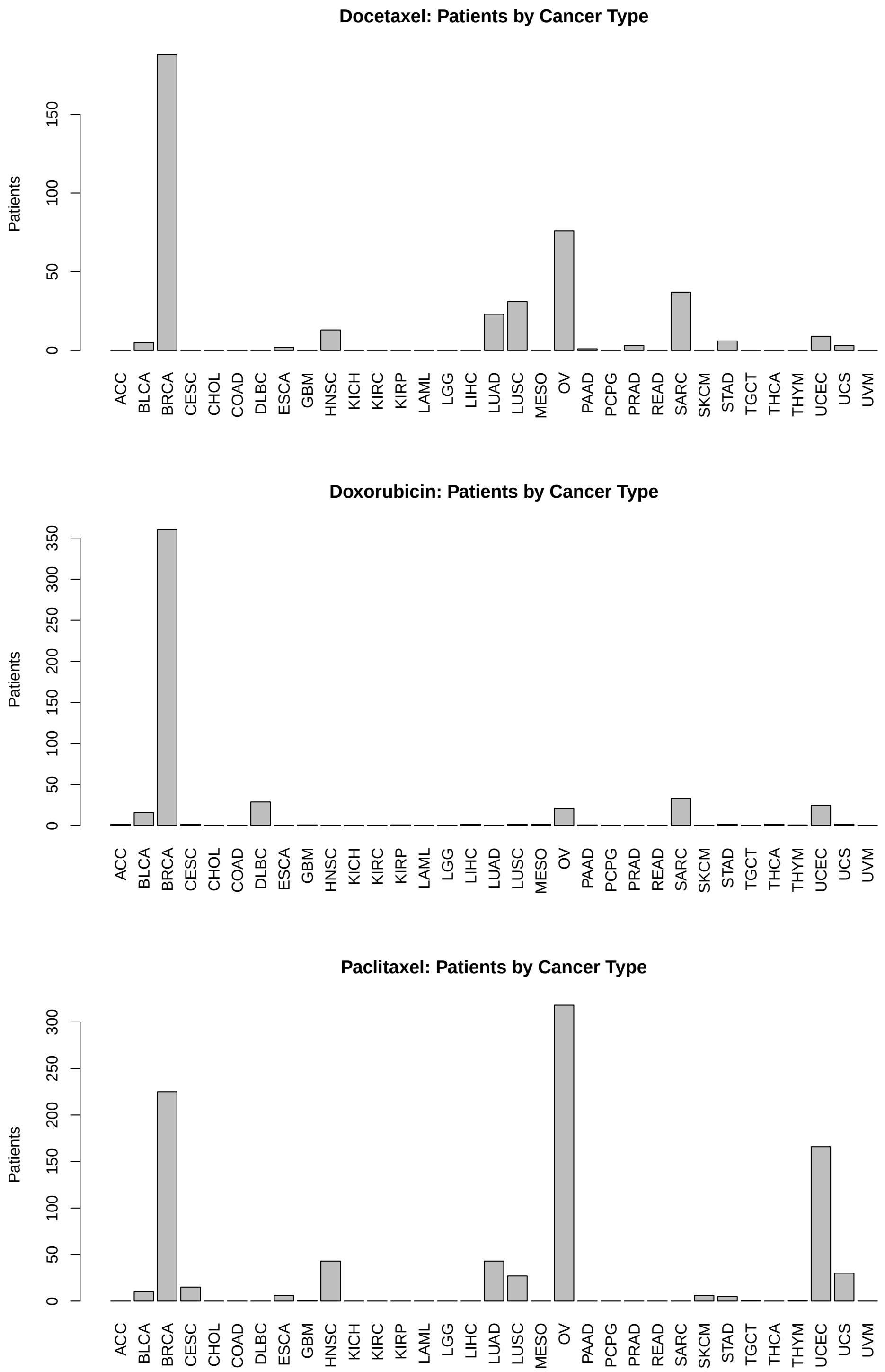

Figure S3. Patient counts by cancer type, for each drug studied in gene expression survival analysis.

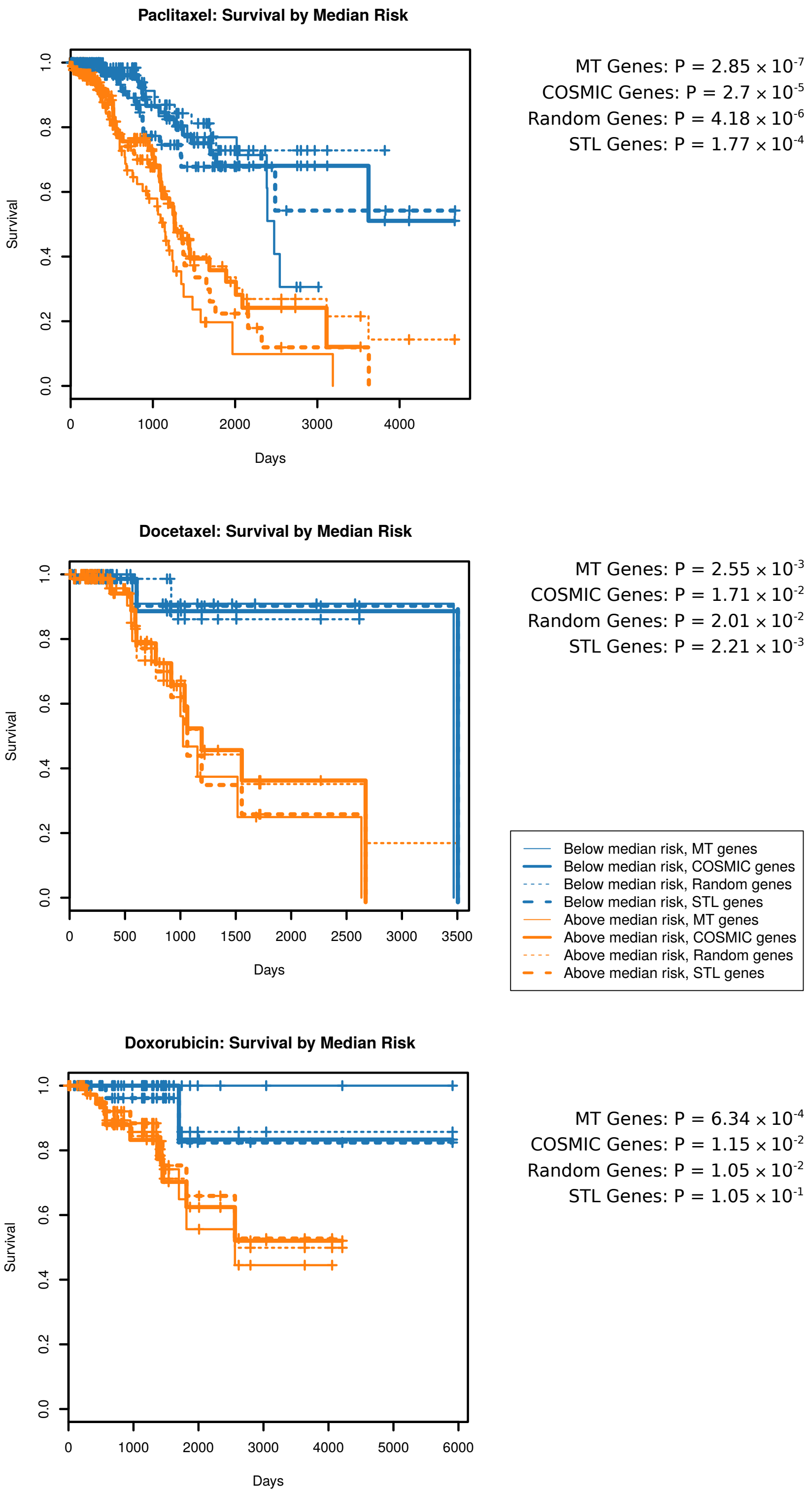

Figure S4. Kaplan-Meier survival curves for patient stratification by median risk in training set samples, for analysis types included in the main text, with curves for each drug included in the same plot for comparison.

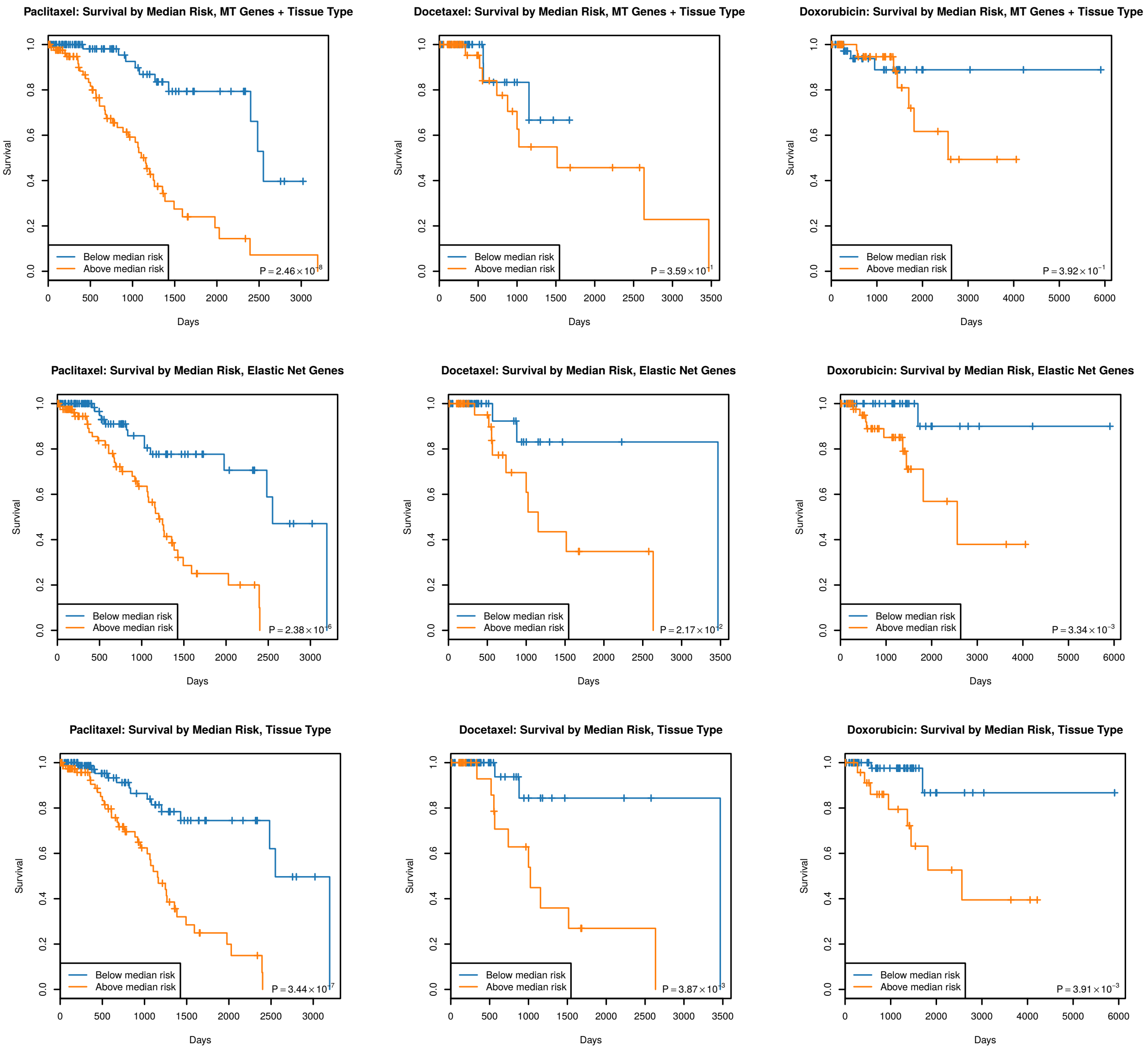

Figure S5. Kaplan-Meier survival curves for patient stratification by median risk in training set samples, for analysis types not included in the main text.

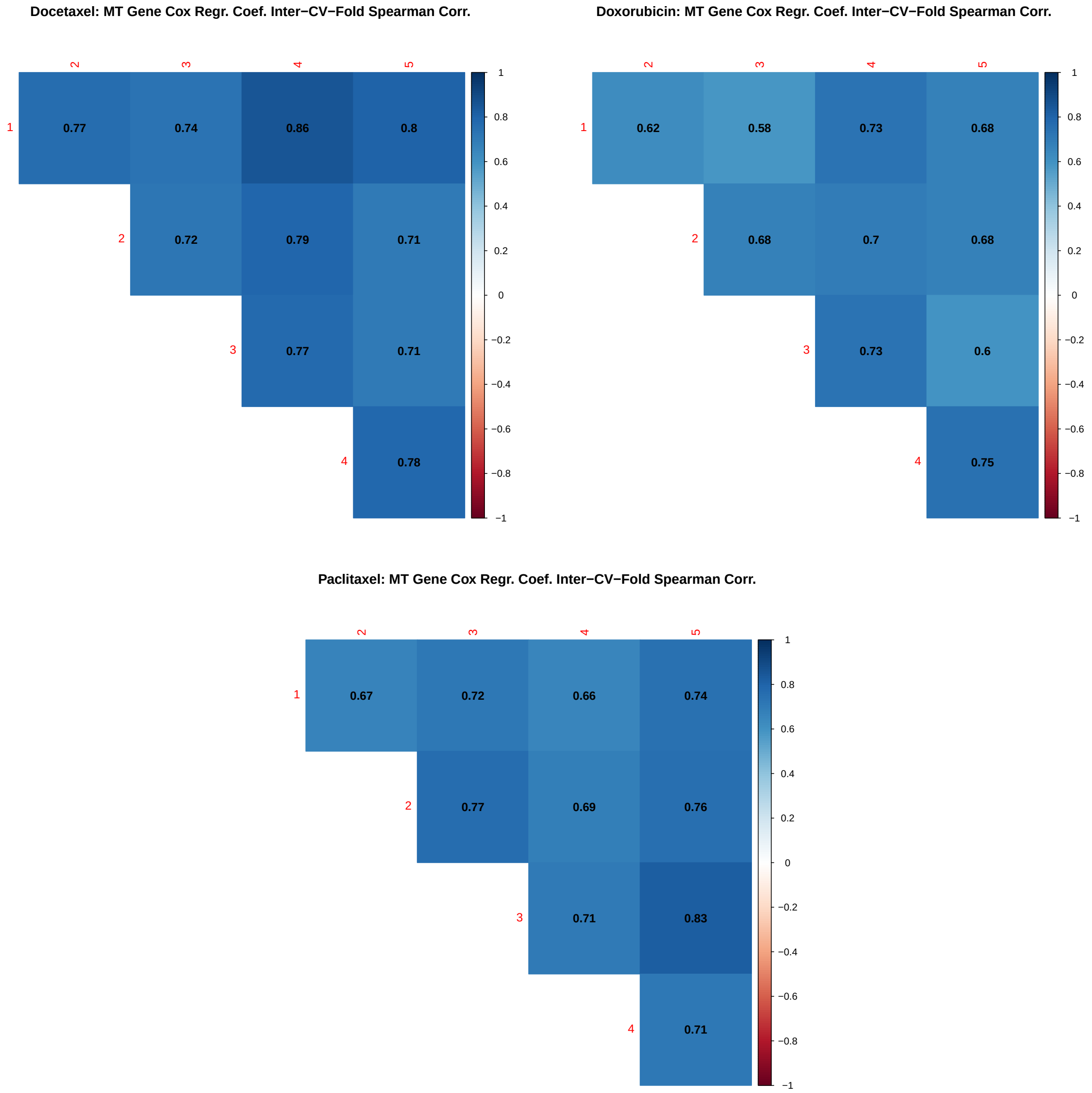

Figure S6. Spearman correlation of MT genes' Cox regression coefficients between 5 cross-validation folds.

8 NDCG Measure

We additionally evaluate our gene rankings using the normalized discounted cumulative gain measure (nDCG) [2]. The nDCG measure is defined on an ordered list of relevance scores, denoted rel_i for item i, and is computed in terms of the “plain” discounted cumulative gain [1] at some position p:

$$\mathrm{DCG}_p = \sum_{i=1}^{p} \frac{2^{rel_i} - 1}{\log_2(i+1)} \tag{1}$$

The nDCG measure normalizes the DCG value, dividing it by the “ideal” DCG value IDCG, obtained from a perfect ranking of the relevance scores under consideration:

$$\mathrm{nDCG}_p = \frac{\mathrm{DCG}_p}{\mathrm{IDCG}_p} \tag{2}$$

where IDCG_p is defined as DCG_p at position p of the same list of relevance scores, but *sorted in descending order.*

We examine our multi-task gene rankings in terms of multiple biologically relevant validation sets: 1,454 gene sets associated with GO terms, 10,295 from MSigDB, and 189 oncogenic gene sets (data posted on the supplementary website). For each validation gene set S, we compute a binary vector v, of length n for the ordered list of genes we identify for each $(\text{drug}, \text{cell line})$ pair, with $v_i = 1$ if gene $i \in S$ and $v_i = 0$ otherwise. We then compute the nDCG measure at position n using binary relevance scores, and we note that $\text{nDCG} = 1$ if and only if the ranking from our multi-task framework has ordered all genes in S above all genes $\notin S$.

For each collection of validation sets (gene sets from: GO terms, MSigDB, oncogenic), we compute the number of validation sets in which we achieve a nDCG value of 1 for each $(\text{drug}, \text{cell line})$ pair (shown in blue bars in Supplementary Figure S7). We perform randomized testing to evaluate the results for our real gene lists: first, we compute the union of all genes in each collection of validation sets, producing three “overall” sets of genes: one for each of $\{\text{GO terms}, \text{MSigDB}, \text{oncogenic}\}$. Then, for each $(\text{overall gene set}, \text{drug}, \text{cell line})$ triplet, we randomly sample k genes from the overall gene set, with k equal to the number of genes identified by our MT method for that $(\text{drug}, \text{cell line})$ pair. We repeat this random sampling 100 times, and for each random sample of gene lists, we again compute the number of times we obtain $\text{nDCG} = 1$ across all gene sets in each validation set collection. We then compute the mean number of times we obtain a perfect nDCG score across all random permutations, with $\mu \pm \sigma$ shown in green bars in Supplementary Figure S7. This figure includes only those results that have more than 10 GO categories, more than 30 MSigDB gene sets, or more than 3 oncogenic sets.

9 Supplementary Tables

Table S2 contains the drugs used in the filtering for high-degree genes. Tables S5, S6, and S7 contain the counts that contribute to the average values (across drugs) shown in Table 1 in the main text. Table S8 shows the counts for each drug contributing to the average values in Table 2 of the main text. For each setting we have overlap statistics for the multi-task scenario (labeled as ”common_census_overlap” for CGC and “genesets_mtl_GO”, “genesets_mtl_oncogenic” for the respective MSIGDB settings), and the single-task scenario: labeled as (“stl_overlap_i” for CGC and “stl_GO_i”, “stl_oncogenic_i” for the MSIGDB genesets, where is the index of the cell type). Each row represents one of the six drugs for each setting.

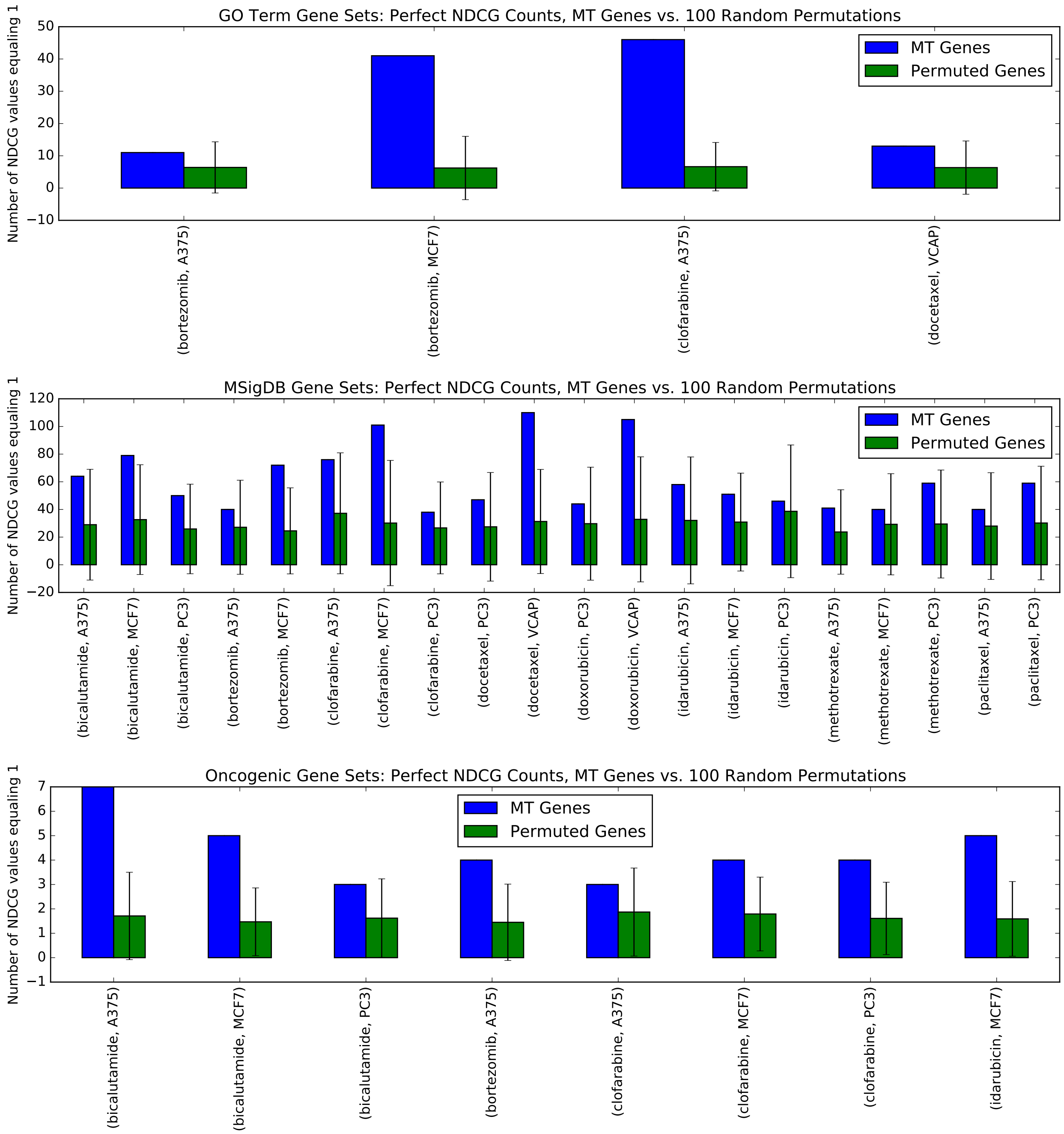

Figure S7. NDCG results: number of validation sets in each category (GO, MSigDB, oncogenic gene sets) in which the gene ranking from our multi-task method produced a normalized discounted cumulative gain measure of 1, vs. same-sized random samples of genes in the corresponding validation sets. Only cell line/drug combinations are shown which had better results for either random rankings rankings, MT gene rankings, or show good performance in both.

References

1. Burges, C., Shaked, T., Renshaw, E., Lazier, A., Deeds, M., Hamilton, N., and Hullender, G. Learning to rank using gradient descent. In *Proceedings of the 22nd international conference on Machine learning* (2005), ACM, pp. 89–96.

2. Järvelin, K., and Kekäläinen, J. Cumulated gain-based evaluation of ir techniques. *ACM Transactions on Information Systems (TOIS) 20*, 4 (2002), 422–446.

**Drug**	**Target**
bicalutamide	AR
	CFLAR
bortezomib	FGFR3
	FLT3
	NFKB1
	PIPSL
	PSMA1
	PSMA2
	PSMA3
	PSMA4
	PSMA5
	PSMA6
	PSMA7
	PSMA8
	PSMB1
	PSMB10
	PSMB2
	PSMB3
	PSMB4
	PSMB5
	PSMB6
	PSMB7
	PSMB8
	PSMB9
	PSMC1
	PSMC1P1
	PSMC2
	PSMC3
	PSMC3IP
	PSMC4
	PSMC5
	PSMC6
	PSMD1
	PSMD10
	PSMD10P1
	PSMD10P2
	PSMD10P3
	PSMD11
	PSMD12
	PSMD13
	PSMD14

Drug	Target
	PSMD2
	PSMD3
	PSMD4
	PSMD4P1
	PSMD5
	PSMD6
	PSMD7
	PSMD8
	PSMD9
	PSME1
	PSME2
	PSME2P2
	PSME3
	PSME4
	PSMF1
clofarabine	FLT3
	POLA1
	RRM1
	RRM2
idarubicin	DNMT3A
	TOP2A
methotrexate	DHFR
paclitaxel	ABCB1
	ABCC10
	ABCC3
	AURKA
	BCL2
	BIRC5
	EGFR
	ERCC2
	GSTP1
	KRAS
	MAP2
	MAP4
	MAPT
	NR1I2
	PDCD4
	PGP
	PTEN
	STMN1
	SYK
	TIMP1
	TLE3
	TLR4
	TOP2A
	TUBB
	TUBB1
	TUBB3

Table S3. Protein targets for drugs studied in this work, according to queries to DGIdb, `http://dgidb.genome.wustl.edu/`.

bicalutamide	bortezomib	clofarabine	idarubicin	methotrexate	paclitaxel
ABCF3	ABCA1	ACLY	ABCA1	ABCG8	ACAT1
ABCG8	ABCB5	ACSL4	ABCC5	ACAD8	ACBD3
ACLY	ACSL6	ACVR1	ACADSB	ADAT1	ACTB
ACO2	ACTR2	AK2	ACSL4	AFF4	ACVR1
ACTB	AES	AKR1B1	AFF4	AFG3L2	ADSS
ACY1	AFG3L2	AKT3	AHR	AKAP13	AKAP17A
AFF4	AHR	ALDH1A1	AK3	AKT1	AKR1B1
AIP	AKR1B1	AMDHD2	AKT2	ALDH1B1	ALDH7A1
ALDH3B1	ALDH3B1	ANXA5	ALDH2	ALX1	ALX1
AP2A2	AMIGO3	APEX1	ALDOA	ANXA2	ANPEP
ARHGAP35	AOC3	ARF4	ANKRD49	APBB2	ANXA5
ASAP2	APOH	ARF6	AOC3	APOB	APOH
ASRGL1	ARG2	ARG1	APRT	APP	APRT
ATP6V0C	ARGLU1	ARHGAP9	ARF4	AR	ARF4
B4GALNT1	ARHGAP35	ARHGEF12	ARHGAP35	ARF4	ASF1B
B4GALT4	ARRB1	ARPC2	ASGR2	ARHGAP1	ASNA1
BACH1	ASNS	ASGR2	ATF6	ARPC4	ATF6
BAK1	ATF6	ATF6	ATP1A3	ATP5D	ATG16L1
BBC3	ATG5	ATP5G1	ATP5F1	ATP5F1	ATIC
BCAT1	ATMIN	ATP6V0C	ATP6AP1	ATP6V1F	ATP5D
BIK	ATP5B	ATXN3	ATXN3	AXL	ATP5L
BUB3	ATP5F1	AURKB	AURKA	B4GALT4	ATP6V1A
BUD13	ATP7A	BAG6	B4GALT4	BAMBI	ATR
C14orf181	AURKA	BCAR1	BAG6	BBC3	*AURKA
C16orf5	AXL	BTG1	BAK1	BCL2L2	AXL
C19orf6	B4GALT4	BUB3	BATF3	BHMT2	AZI2
CA12	BCCIP	BUD31	BLM	BLM	BACH1
CARD10	BIRC6	C19orf6	BLVRA	BLVRB	BBC3
CASP6	BLM	C2CD2	BRAP	BRP44	BBS9
CBFA2T3	BMPR1A	CALM1	BTG3	BRPF3	BCL2L11
CCDC90A	BRPF3	CAMSAP2	C19orf6	CALR	BCL9
CCNF	C14orf181	CAPNS2	C1QTNF6	CANT1	BIRC2
CDC25A	C19orf6	CASP9	CALR	CBX6	BLVRB
CDKN1C	C1QTNF6	CBLB	CAMSAP2	CCND1	BMP2
CEBPD	CALM1	CBS	CAPNS2	CCT8	BRAF
CEPT1	CAMK2D	CCL2	CBR3	CD24	BRF2
CES2	CAT	CCNB1	CBX6	CD3D	BRPF1
CGRRF1	CCNA1	CCT7	CCL2	CD58	C19orf6
CHD1	CCNC	CCT8	CCND3	CDK11B	CALM3
CLSTN1	CCNH	CD40	CCNH	CDK4	CCNG1
CLTA	CCT7	CDC42SE1	CCT7	CDK8	CCT8
CNOT8	CD44	CDH1	CD83	CEBPZ	CD19
COX5B	CDC42	CDKN2C	CDC25A	CELSR2	CD1C
CS	CDCA7L	CEBPG	CIITA	CENPE	CD24
CSAD	CDK11B	CFTR	CLPB	CLK2	CD81
CSNK1D	CEBPG	CHERP	CNOT3	COPA	CD99
CTSL1	CEBPZ	CHN1	CNOT8	COPZ1	CDK1
CXCR2	CEPT1	CHRM1	COG4	CPE	CDX1
CXXC4	CERS3	CLCN3	COL13A1	CREB3L4	CEACAM1
CYTH1	CHERP	CLPB	COPZ1	CSNK1G2	CHRAC1

bicalutamide	bortezomib	clofarabine	idarubicin	methotrexate	paclitaxel
DACH2	CHRAC1	CNOT3	CRKL	CTBP1	CLK3
DARS	CNOT3	COL4A5	CS	CTH	CLN3
DPY30	COG4	COPS2	CSNK1G2	CTNNAL1	CLOCK
DROSHA	COPA	COPS5	CTCF	CTSB	COPS5
EBNA1BP2	COPS2	CRADD	CTPS	CYB5A	COPZ1
EFNB2	COPZ1	CSNK1E	CTSL1	DDIT3	CRABP2
EGR3	CRADD	CSNK2B	CXCL1	DEK	CRADD
EHHADH	CREM	CSRP1	DCLRE1B	DGKD	CSDA
EIF3H	CSNK1A1	CTBP1	DCPS	DNAJA3	CTBP1
EIF6	CSNK1E	CYP51A1	DEK	DNPEP	CTSK
EMD	CXCL1	DARS	DLX2	DYRK1A	CXCR7
ENPP1	DDX49	DCLRE1B	DMTF1	ELF4	CYP27B1
ENTPD6	DDX5	DDX42	DUT	EP400	DGKZ
EPHA3	DGKD	DDX5	DYNLL2	ERCC1	DHX8
ERCC1	DGKZ	DERA	EIF2AK1	ESR1	DLC1
ERGIC2	EAPP	DNTTIP2	ELL3	EXOC6	DLX3
ERRFI1	EIF2B2	DYNLL2	EPC1	FADS1	DMD
ETNK1	EIF2B3	E2F5	ERBB3	FAM102A	DNAJB12
ETV4	EIF3H	EBNA1BP2	ERGIC1	FASLG	DNMT3L
ETV5	EP300	ECHS1	ESR1	FAT1	DPF1
F11	EPHB4	EIF2B2	ETS1	FCGR2A	EFNB2
FABP5	ERBB3	EMR1	EXOC6	FGG	EIF2S2
FASN	ERGIC2	ERCC5	FADS3	FKBP8	ELF1
FBXL12	ESR2	ERF	FCGR2A	FRAT1	ENDOG
FDX1L	EXOSC4	ERGIC1	FECH	FUT2	ESD
FGG	FAS	EXOSC4	FGG	FZD7	ESYT1
FRAT1	FGFR4	FADS1	FOS	GALC	EXOC2
FZD7	FLI1	FGFR1	FRAT1	GATAD1	F3
FZD8	FOS	FOSL2	FZD4	GDI1	F7
G2E3	FZD4	GALC	FZD7	GGCX	FBXO11
G6PC	FZD8	GATAD1	FZD8	GGH	FDX1L
GABRA5	G2E3	GMNN	GALC	GIT1	FEN1
GART	GABRA1	GNG4	GATAD1	GMDS	FERD3L
GCK	GADD45B	GNG5	GBP2	GMPS	FGFR1OP
GFOD2	GBP2	GPER	GCAT	GNA12	FKBP8
GFPT2	GCK	GPR39	GLI1	GPR111	FOXA3
GJA1	GLA	GPR78	GMPR2	GPR141	FRS2
GLRX	GLI1	GSK3A	GMPS	GPR31	FZD6
GNA13	GPER	GTF2A2	GNA15	GSDMB	GABPB1
GNB5	GPR110	HADH	GNE	HAL	GAMT
GPI	GPR111	HADHB	GPR107	HBE1	GATAD1
GPR128	GPR115	HCFC2	GPR115	HCAR1	GBP2
GPR152	GPR156	HDHD1	GPR156	HCFC2	GCLC
GPR87	GPR39	HEATR1	GPR78	HDAC3	GNAS
GPX7	GPR62	HFE	GRB7	HERPUD1	GPR111
GTF2E2	GPR78	HOXC9	GTF2A1	HIST1H1E	GPR112
HES5	HGS	HPD	GTF2H3	HIST1H2BK	GPR176
HLA-DMA	HIF1AN	HSPG2	HBE1	HK1	GPR39
HMGCS2	HIST1H1B	IFNG	HIBADH	HLA-DRB4	GPR64
HMGN4	HLA-DRB5	IGF2BP1	HIST1H2BK	HNF4G	GPR83

bicalutamide	bortezomib	clofarabine	idarubicin	methotrexate	paclitaxel
HNF4G	HMGB1	IGFBP2	HMGB1	HOXA1	GPRC6A
HOMEZ	HMGN4	IGHMBP2	HMGB4	HOXC10	GPX2
HOXB13	HOXA10	IL23R	HMGCS1	HS2ST1	GPX3
HOXC10	HOXC10	ILF2	HMMR	HSD17B10	GSS
HRH1	HSF5	IRAK2	HOXB4	HSPD1	*GSTP1
HSPA9	HSP90AB1	ISL1	HOXC10	HSPE1	GTF2F2
IARS2	HSPA5	ITPK1	HSPB1	HTATSF1	HAT1
IDH3B	IKBKAP	KAT2B	HSPD1	HTRA1	HGS
IL18	IL13	KIAA1033	ICAM1	ICAM1	HIST1H2BD
INPP4B	IL20	KIF2C	IL23R	IDH3B	HIST2H2BE
IPMK	IL6ST	KLF6	IL6ST	IFNG	HMGCS1
ITGA2	INPP5D	LHX4	IMPA1	IL1B	HMOX1
ITGA4	IPO4	LIAS	IRAK2	IMPDH1	HOOK2
KCNK2	IRS2	LIN28A	ISG20	INPP5D	HOPX
KIAA0100	ISG20	LPL	ITGA2	INS	HSF1
KRAS	ITGAV	LRSAM1	ITGAV	IPO4	IDH1
KRT8	ITGB1BP1	LYPD3	ITPR1	IQGAP1	IGFBP3
KSR2	JAG1	LYZ	JAG1	IRF4	ILF2
L1CAM	KARS	MAT2A	KARS	ISL1	IPMK
LANCL1	KEAP1	MBNL1	KCNK1	ITGA3	IPO4
LARS	KLRC1	MCM3	KCNK15	ITPR1	IQGAP1
LIPA	LAGE3	MCM6	KDELR3	JAZF1	IRAK2
LONRF2	LGR6	MECP2	KDM3A	KAT7	IRS2
LPXN	LIG1	MED1	KLRC1	KLF3	ISL1
LRPPRC	LRP5	MEOX2	KRAS	KREMEN1	ITGB3
LSM5	LRPPRC	MFSD10	LGR6	LAGE3	ITPKA
LTA4H	LTBR	MGAT1	LHX4	LARS2	KAT2B
MAFG	LZIC	MGST2	LIN28A	LGALS2	KAZALD1
MAOB	MAD2L1BP	MKI67IP	LRSAM1	LHX2	KDELR3
MAP2K4	MAP3K8	MLL4	LZIC	LOXL1	KIAA0753
MAP2K6	MAPK1	MRGPRF	MAP3K8	LPXN	KIF11
MAPKAP1	MARK4	MRPL18	MAPKAPK5	LRP10	KIF13B
MAPKAPK5	MCM7	MTA1	MAS1L	LRRC59	KITLG
MCM2	MED11	MTF2	MATN3	LRSAM1	KLF11
MCM7	MGAT1	MTFR1	MDM2	LSS	KLF14
MED28	MLEC	MTR	MEOX2	MAP2K1	KLF4
METTL14	MOS	MYC	MGLL	MAP3K2	KRT25
MLLT6	MPL	MYL9	MLEC	MAP3K7	LAMP2
MUT	MTA1	NAGA	MTFR1	MAPK1	LEF1
MYD88	MTF2	NDUFS1	MYBL2	MAPKAPK5	LGALS3
MYL6	MVP	NDUFS8	MYLK	MARK4	LGR5
MYL9	MXD4	NFKB1	NAE1	MAT1A	LPAR3
NCOA2	MYBL2	NFKBIL1	NARS	MAT2A	LTA4H
NDUFA2	NAB1	NIT1	NDUFA1	MB	LYPLA1
NDUFA4	NAGPA	NME7	NDUFA4	MEOX2	MAP2K4
NDUFA5	NCL	NOS3	NDUFAF4	MGAT1	MARS
NDUFB7	NDUFA5	NPTN	NDUFS1	MGLL	MAST4
NDUFS6	NDUFA6	NUP62	NDUFS3	MITF	MCOLN1
NDUFS8	NDUFS6	NVL	NDUFS6	MKNK2	MDM2
NEDD4	NFKBIL1	NXF1	NEK7	MLEC	MFAP3

bicalutamide	bortezomib	clofarabine	idarubicin	methotrexate	paclitaxel
NFIL3	NIT1	PAF1	NFIB	MLL	MGMT
NGFR	NLK	PDHX	NIT1	MLLT10	MIXL1
NKX2-5	NME4	PDS5B	NME7	MYB	MLL3
NNT	NMUR2	PET112	NNMT	MYCN	MLL4
NOX1	NUDT9	PFAS	NOTCH2NL	NAB1	MPHOSPH9
NPBWR1	NXF1	PHB2	NR0B2	NAGA	MRGPRX3
NQO1	OAZ2	PHF13	NT5C2	NCL	MSH6
NSDHL	OPA1	PIK3CD	NUDT6	NCOR2	MSRA
NT5C2	PARN	PIK3R4	NUDT9	NDUFA1	MST1R
ODC1	PCNA	PMF1	NUMB	NDUFA5	MYBL2
OGG1	PHACTR1	PNKP	NVL	NDUFB2	MYCL2
OSGEP	PHB2	POLE2	NXF1	NEK7	MYLK2
P2RY6	PHF16	POLR1A	ORC1	NFE2L1	MYO10
PAK1	PHF21B	PPAP2B	OSMR	NFKB1	NAA50
PAPD7	PLA2G4A	PPIE	OTUD7A	NFKBIB	NDUFB5
PEX13	PMPCB	PPM1D	OXGR1	NIPBL	NDUFC2
PEX19	PNN	PPP1CA	P4HA1	NKX2-3	NDUFS4
PFAS	POFUT1	PPP2R4	P4HB	NQO1	NDUFV2
PIK3CG	POLR2A	PPP4R1	PAPOLA	NR0B2	NEU1
PIK3R1	POLR2I	PQBP1	PARN	NR2F2	NGFR
PIM2	PPAP2B	PRCP	PDGFRB	NRIP1	NIT1
PIM3	PPFIBP2	PREB	PHF21B	NUMB	NMUR2
PJA2	PPIE	PROKR2	PI4KAP2	NUP88	NOD1
PLA2G15	PPIH	PRSS42	PIK3CG	OGG1	NONO
PNPO	PPP1R13B	PSMA3	PIK3R1	P4HB	NPFFR2
POLR2F	PPP1R14B	PSMD2	PJA2	PAPOLA	NUCKS1
POLR3B	PPP2R5C	PSMD3	PLD2	PCBD1	NXF1
POLR3E	PRCP	PSPH	POFUT1	PCNA	P2RY8
PON2	PREB	PSRC1	POLR2A	PDHX	P4HTM
PPP3CB	PRKAR1B	PTEN	POLR2H	PDPK1	PAF1
PPP4R1	*PSMA1	PTGER4	POLR2I	PDS5B	PAFAH1B2
PRDX5	*PSMA3	PTGR1	POLR3F	PGD	PAN2
PRPSAP2	*PSMA7	PTHLH	PPAP2B	PHB	PAPD7
PRSS23	*PSMB1	PTMS	PPFIBP2	PHF23	PASD1
PSMA3	*PSMB2	PTPN6	PPP1R14B	PI4KAP2	PBXIP1
PSMB5	*PSMB5	PURA	PPP3CC	PIAS4	PDE4D
PTK2B	*PSMB7	Pparg	PQBP1	PIK3R4	PEBP1
PTPN4	*PSMD1	RAB11A	PRKAG3	PJA2	PEMT
PTRF	*PSMD2	RAB5A	PRSS3	PKN2	PIAS3
PTTG1	*PSMD3	RAD51	PRSS42	PLAT	PIGB
PUF60	*PSMD8	RAD51C	PSEN1	POLA2	PKIA
RAB5A	PTMS	RALA	PSMB1	POLB	PLA2G2A
RAI14	PUF60	RAN	PSMB10	POLE2	PLK2
RALB	PVALB	RBBP7	PSMB2	POLR2A	PLOD3
RASD1	PYCRL	RBL2	PSMD1	POLR2C	PMF1
RBL1	Pparg	REL	PSMD8	POLR2I	PNKP
RBMX	RAB5A	RHOB	PSPH	PPAP2B	PPAP2A
RFC5	RAN	RIOK2	PXK	PPIE	PPAP2B
RNASE4	RASSF2	RIPK2	RAC1	PQBP1	PPIB
RNF167	RBP1	RNF7	RASGRP4	PRDX1	PPIE

bicalutamide	bortezomib	clofarabine	idarubicin	methotrexate	paclitaxel
RNF5	RBX1	RPA1	RASSF2	PRKAR1B	PPOX
RPS16	RIPK1	RPA2	RBL2	PROC	PRKAA1
RPS6KB2	RIPK3	RPN1	RHOB	PRSS3	PRKCE
RRP12	RNF125	RPN2	RIOK2	PRSS42	PRKCZ
RUNX1	RNPS1	RPS6	RIPK1	PSAP	PRMT2
RUVBL2	RPN2	RPS9	RIPK3	PSMB2	PROKR1
RXRA	RPS14	RRAGB	RNF138	PSMD1	PRPF4B
SAR1B	RPS15A	*RRM1	RPN1	PTGR1	PSMA5
SATB1	RPS16	RRP1B	RPN2	PTPN1	PSMA8
SBNO1	RPS27A	RRP8	RPS14	PTPN6	PSMB5
SEC24B	RPS6	RRS1	RPS15A	PYCRL	PSMB8
SERINC3	RPS6KB2	RUVBL1	RPS16	QARS	PSMD2
SERPINF2	RPS9	SACM1L	RPS19	QPRT	PSMD3
SHC4	RRAGD	SCMH1	RPS27A	RAB5A	PSMD8
SIAH1	RRM1	SCUBE1	RPS3	RAB7A	PTMS
SIK3	RUVBL1	SCYL3	RPS3A	RASA1	PTPN6
SIX2	RUVBL2	SENP6	RPS6	RBCK1	RAB23
SLC16A1	RYK	SET	RPS9	RIPK1	RAB4A
SLC25A14	SAFB	SF1	RRM1	RNF133	RAB5B
SLC36A1	SCMH1	SGCB	RUVBL1	RPS15A	RAN
SLC37A4	SDHA	SH3BP5	RUVBL2	RPS16	RAP1GAP
SMARCA2	SEC16A	SIN3A	RXRG	RPS19	RASD1
SMNDC1	SEC24D	SIRT7	SACM1L	RPS27A	RBCK1
SOAT2	SEC61A2	SLC16A5	SCAF8	RPS3A	RBFOX3
SOCS4	SERINC3	SLC25A4	SDHA	RPS6	RDH11
SPHK2	SERPINA1	SLC25A5	SDHD	RPS9	RDX
SPIC	SFPQ	SLC2A6	SERPINA7	RPTN	REL
SRM	SLC16A5	SLC6A14	SLC16A1	RRAGB	RELB
SRPK1	SMARCC1	SMC3	SLC16A5	RRM1	RFC2
SSBP2	SOX2	SNW1	SLIRP	RUVBL1	RFXANK
ST14	SPTAN1	SNX2	SMAD1	SEC24D	RGS2
STAMBP	SRRT	SPP1	SMAD4	SET	RHOBTB1
STK39	SRSF3	SPR	SMAD7	SHC4	RIPK3
STUB1	STK38	SPTAN1	SMOC2	SKP2	RNF123
SUPT3H	STMN1	SRPRB	SNCA	SLC16A1	RNF167
SYNGR3	SULT1A3	STAMBP	SOD1	SLC25A4	RNPS1
TAB2	SYT1	STAT1	SOX2	SLC25A6	ROS1
TAF15	TBX20	STAT3	SPDEF	SLC2A6	RPN2
TAL2	TERF1	STMN1	SPP1	SLC39A6	RPS27A
TBC1D2B	TK1	STX1A	SPRY2	SLC9A1	RPS6
TBL3	TLE1	SULT1A3	SPRY4	SLIRP	RUNX1
TCIRG1	TMED10	SULT2A1	STK16	SMOC2	SACM1L
TGDS	TNFRSF13C	SUZ12	STK39	SNCA	SCAF8
TH	TOLLIP	SYNE2	STMN1	SOD1	SCCPDH
TIMM50	TRAM2	TCERG1	SULT1A3	SOX10	SCMH1
TJP1	TRAPPC3	TERF1	SUPT5H	SPECC1L	SDHB
TLR2	TRIM13	TFAP2A	SUPV3L1	SPR	SERPINF2
TM9SF2	TRIM27	TIPARP	SUZ12	SPRED2	SF3B1
TMEM97	TRPS1	TM9SF2	TAB3	SPRY2	SFN
TNFRSF10A	TXNL4B	TMED10	TARDBP	SRPK1	SKIV2L

bicalutamide	bortezomib	clofarabine	idarubicin	methotrexate	paclitaxel
TNFSF10	TXNRD1	TNFRSF12A	TERF1	STAMBP	SKP2
TNFSF4	UBC	TOMM70A	TESK1	STAT6	SLC25A1
TNIK	UIMC1	TOPBP1	TIPARP	STK16	SLC25A24
TNPO3	ULK3	TUBB3	TLE1	STK39	SLC38A3
TP53RK	UQCRC1	TYMS	TNFRSF13C	STMN1	SLC3A1
TPR	UROD	UBA52	TNFRSF18	SULT1A3	SMARCC2
TPRKB	VAMP3	UBE2V1	TOLLIP	TAAR6	SMC3
TRAF3	VCP	UBE2Z	TOMM40	TAB2	SMO
TRAF6	VDAC1	UIMC1	TRAF6	TAB3	SNAPC1
TRAP1	VIM	UQCRFS1	UBC	TACC3	SOCS4
TXNDC9	VPS28	USP14	UGT2B28	TANK	SORT1
TXNIP	VRK1	USP7	ULK3	TARDBP	SQLE
UBB	WARS2	UTP18	USP7	TCERG1	SRGAP2
UBE2J1	XBP1	VAMP3	UTP18	TERF1	SUCLG2
UBE2K	XIAP	VCP	VAMP3	TFF2	TDRD3
UBE4A	ZMYM2	VPS28	VCAN	TIMM22	TERF1
UGT1A3	ZNF175	WARS2	VCP	TMED10	TFF2
ULK3	ZNF562	WNT1	VIM	TP53RK	TINF2
USP15	ZNF596	XRCC5	VRK1	TPD52L3	TMED10
USP22	ZNF626	YAF2	YME1L1	TRAF1	TRAPPC3
USP9X	ZNF717	ZBTB45	YTHDF1	TSC22D1	TSPAN3
VN1R2	ZNF785	ZBTB48	ZFAND6	TSG101	TSPAN4
WEE1		ZFP112	ZFP3	TSKU	TSTA3
YME1L1		ZMYM2	ZFP36L2	TSPAN4	*TUBB3
ZFAND6		ZMYND11	ZNF114	TST	TXNL4B
ZMYND11		ZNF238	ZNF174	TUBD1	UBE2D3
ZNF140		ZNF318	ZNF22	UBAP2L	UNC5D
ZNF296		ZNF385B	ZNF398	UBC	UQCR11
ZNF32		ZNF449	ZNF596	UBE2D1	USP20
ZNF436		ZNF608	ZNF668	UNC13B	USP7
ZNF57		ZNF653	ZNF689	VAMP3	VAV3
ZNF581		ZNF678	ZNF785	VPS28	VCP
ZNF623		ZNF785		WARS	VIM
ZNF662		ZNF786		WARS2	WARS2
ZNF673		ZNHIT3		WNT9B	ZIM3
ZNF684				WRN	ZNF133
				YWHAZ	ZNF238
				YY1	ZNF267
				ZMYND11	ZNF395
				ZNF22	ZNF543
				ZNF623	ZNF678
				ZNF658	ZNF74
					ZNF791
					ZSWIM2

Table S4. Drug targets used in this work, computed via gene expression correlation with LINCS data. Gene names are prefixed with "*" if that gene is also included in the DGIdb data for the respective drug.

CGC	common_census_overlap	stl_overlap_Breast	stl_overlap_Prostate
Methotrexate	15	14	12
Clofarabine	15	8	7
Idarubicin	15	10	7
Paclitaxel	15	5	12
Bicalutamide	13	6	7
Bortezomib	15	7	15
**average**	14.66	8.33	10
GO Genesets	genesets_mtl_GO	stl_GO_Breast	stl_GO_Prostate
Methotrexate	73	92	70
Clofarabine	76	61	63
Idarubicin	75	73	48
Paclitaxel	68	60	48
Bicalutamide	74	63	67
Bortezomib	99	75	93
**average**	77.5	70.66	64.83
Oncogenic	genesets_mtl_oncogenic	stl_oncogenic_Breast	stl_oncogenic_Prostate
Methotrexate	14	8	2
Clofarabine	12	0	7
Idarubicin	9	2	6
Paclitaxel	2	7	4
Bicalutamide	2	2	6
Bortezomib	13	7	6
**average**	8.66	4.33	5.166

Table S5. Statistical Results for Breast and Prostate Cancer

CGC	common_census_overlap	stl_overlap_PC3	stl_overlap_VCAP
Methotrexate	13	13	7
Clofarabine	19	13	12
Idarubicin	13	8	13
Paclitaxel	19	12	11
Bicalutamide	14	9	9
Bortezomib	12	6	10
**average**	15	10.16	10.33
GO Genesets	genesets_mtl_GO	stl_GO_PC3	stl_GO_VCAP
Methotrexate	106	89	62
Clofarabine	62	60	82
Idarubicin	83	83	108
Paclitaxel	108	105	100
Bicalutamide	65	108	92
Bortezomib	70	70	88
**average**	82.33	85.83	88.66
Oncogenic	genesets_mtl_oncogenic	stl_oncogenic_PC3	stl_oncogenic_VCAP
Methotrexate	10	9	6
Clofarabine	11	11	9
Idarubicin	8	4	5
Paclitaxel	20	9	18
Bicalutamide	8	8	4
Bortezomib	9	9	4
**average**	11	8.33	7.66

Table S6. Statistical Results for Only Prostate Cancer

CGC	common_census_overlap	stl_overlap_HA1E	stl_overlap_NPC
Methotrexate	12	7	9
Clofarabine	14	9	8
Idarubicin	11	9	7
Paclitaxel	18	11	9
Bicalutamide	12	6	12
Bortezomib	13	9	7
**average**	13.33	8.5	8.66
GO Genesets	genesets_mtl_GO	stl_GO_HA1E	stl_GO_NPC
Methotrexate	71	44	29
Clofarabine	72	39	21
Idarubicin	66	14	30
Paclitaxel	102	48	52
Bicalutamide	70	23	67
Bortezomib	53	34	50
**average**	72.33	33.66	41.5
Oncogenic	genesets_mtl_oncogenic	stl_oncogenic_HA1E	stl_oncogenic_NPC
Methotrexate	7	3	2
Clofarabine	7	6	2
Idarubicin	7	3	5
Paclitaxel	10	1	5
Bicalutamide	7	3	3
Bortezomib	8	2	3
**average**	7.66	3	3.33

Table S7. Statistical Results for Non-Cancer Cell Types

**CGC**	**MTL__census__overlap**	**STL__overlap__br**	**STL__overlap__pr**	**STL__overlap__mel**
Methotrexate	20	21	23	14
Clofarabine	27	23	28	19
Idarubicin	30	21	28	23
Paclitaxel	33	23	24	24
Bicalutamide	29	21	28	25
Bortezomib	33	25	28	28
**average**	28.66	22.33	22.6	22.16
**GO**	**genesets__MTL__GO**	**STL__GO__br**	**STL__GO__pr**	**STL__GO__mel**
Methotrexate	171	129	200	113
Clofarabine	217	165	218	156
Idarubicin	204	197	232	214
Paclitaxel	256	152	223	251
Bicalutamide	243	233	207	181
Bortezomib	241	202	177	223
**average**	222	179.66	209.66	189.66
**Oncogenic**	**genesets__MTL__oncogenic**	**STL__oncogenic__br**	**STL__oncogenic__pr**	**STL__oncogenic__me**
Methotrexate	7	0	9	1
Clofarabine	14	6	11	5
Idarubicin	2	4	17	2
Paclitaxel	17	5	17	6
Bicalutamide	15	15	16	3
Bortezomib	30	26	19	7
**average**	14.16	9.33	14.83	4

Table S8. Results for Breast Cancer, Prostate Cancer and Melanoma. The abbreviations are: ”br” for breast cancer, ”pr” for prostate cancer and ”mel” for melanoma.

**CGC**	**MTL__census__overlap**	**STL__overlap__br**	**STL__overlap__pr**	**STL__overlap__mel**
Methotrexate	28	22	2	19
Clofarabine	28	24	22	20
Idarubicin	27	22	21	21
Paclitaxel	25	21	21	19
Bicalutamide	25	19	22	22
Bortezomib	32	18	21	19
**average**	27.5	21	21.33	20
**GO**	**genesets__MTL__GO**	**STL__GO__br**	**STL__GO__pr**	**STL__GO__mel**
Methotrexate	225	200	212	167
Clofarabine	218	173	184	213
Idarubicin	247	210	238	202
Paclitaxel	245	182	221	214
Bicalutamide	220	255	193	171
Bortezomib	242	218	223	196
**average**	232.83	206.33	211.83	193.83
**Oncogenic**	**genesets__MTL__oncogenic**	**STL__oncogenic__br**	**STL__oncogenic__pr**	**STL__oncogenic__me**
Methotrexate	16	12	6	5
Clofarabine	14	3	20	19
Idarubicin	10	14	15	5
Paclitaxel	3	0	5	0
Bicalutamide	11	2	8	4
Bortezomib	11	15	16	13
**average**	10.83	7.66	11.66	7.66

Table S9. Results for Breast Cancer, Prostate Cancer and Melanoma with $k = 50$ (where k is the number of sources). The abbreviations are: "br" for breast cancer, "pr" for prostate cancer and "mel" for melanoma.

**Drug \Cell Line**	A375	HA1E	MCF7	PC3	VCAP	**Total**
Bicalutamide	6	7	21	22	31	87
Bortezomib	10	9	60	30	9	118
Clofarabine	5	5	7	10	12	39
Disulfiram	2	2	11	11	10	36
Docetaxel	6	5	19	23	19	72
Doxorubicin	30	46	104	52	15	247
Idarubicin	12	11	78	17	12	130
Ketoconazole	8	5	13	16	20	62
Metformin	77	3	16	13	10	119
Methotrexate	3	27	42	13	10	95
Paclitaxel	9	45	57	49	22	182
Vinblastine	8	37	42	19	18	124
**Total**	176	202	470	275	188	1311

Table S10. LINCS L1000 expression experiment counts by drug and cell line, for drugs and cell lines studied in this work.

Drug Name	LINCS Perturbagen ID	'trt_cp' Experiment Count
Bicalutamide	BRD-A29485665	125
Bortezomib	BRD-K50691590	108
Bortezomib	BRD-K88510285	184
Clofarabine	BRD-A82371568	69
Disulfiram	BRD-K32744045	46
Docetaxel	BRD-K30618791	11
Docetaxel	BRD-K42125900	64
Docetaxel	BRD-K63265447	37
Doxorubicin	BRD-A52530684	200
Doxorubicin	BRD-A76941896	54
Doxorubicin	BRD-K92093830	190
Idarubicin	BRD-A71390734	283
Idarubicin	BRD-K69650333	29
Ketoconazole	BRD-A76019558	44
Ketoconazole	BRD-K29113274	33
Metformin	BRD-K79602928	222
Methotrexate	BRD-A55424491	73
Methotrexate	BRD-K59456551	46
Paclitaxel	BRD-A23723433	229
Paclitaxel	BRD-A28746609	83
Paclitaxel	BRD-K86858124	10
Vinblastine	BRD-A22783572	69
Vinblastine	BRD-A55594068	85
Vinblastine	BRD-A85648045	26
Vinblastine	BRD-K01188359	35

Table S11. Number of experiments per drug, separated by LINCS perturbagen ID – drugs are assigned multiple perturbagen IDs if produced by multiple manufacturers. Experiment counts include *all* cell lines, not only those used in this work. Note that none of the listed perturbagens were used in any experiment types other than 'trt_cp'.

Drug	Patient Count
Bicalutamide	28
Bortezomib	5
Docetaxel	442
Doxorubicin	522
Ketoconazole	2
Metformin	1
Methotrexate	50
Paclitaxel	1071
Vinblastine	23

Table S12. Number of TCGA patients (from a total of $11,159$) prescribed each of the drugs described in the main text.

**Drug**	**Gene Count**
Bicalutamide	94
Bortezomib	120
Clofarabine	112
Docetaxel	65
Doxorubicin	70
Idarubicin	101
Methotrexate	123
Paclitaxel	113

Table S13. Number of genes identified by the multi-task learning framework for drugs studied.
